# Toward a Consistent Prediction of Defect Chemistry in CeO_2_

**DOI:** 10.1021/acs.chemmater.2c03019

**Published:** 2022-12-21

**Authors:** Xingfan Zhang, Lei Zhu, Qing Hou, Jingcheng Guan, You Lu, Thomas W. Keal, John Buckeridge, C. Richard A. Catlow, Alexey A. Sokol

**Affiliations:** † Kathleen Lonsdale Materials Chemistry, Department of Chemistry, University College London, LondonWC1H 0AJ, United Kingdom; ‡ Scientific Computing Department, STFC Daresbury Laboratory, Warrington, CheshireWA4 4AD, United Kingdom; § School of Engineering, London South Bank University, LondonSE1 OAA, United Kingdom; ∥ School of Chemistry, Cardiff University, Park Place, CardiffCF10 1AT, United Kingdom; ⊥ Institute of Photonic Chips, University of Shanghai for Science and Technology, Shanghai200093, China

## Abstract

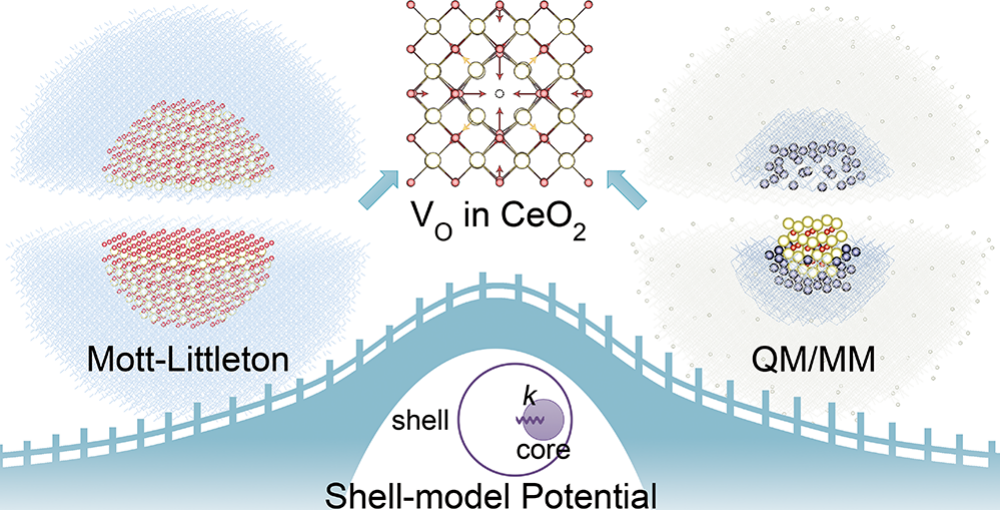

Polarizable shell-model potentials are widely used for atomic-scale modeling of charged defects in solids using the Mott–Littleton approach and hybrid Quantum Mechanical/Molecular Mechanical (QM/MM) embedded-cluster techniques. However, at the pure MM level of theory, the calculated defect energetics may not satisfy the requirement of quantitative predictions and are limited to only certain charged states. Here, we proposed a novel interatomic potential development scheme that unifies the predictions of all relevant charged defects in CeO_2_ based on the Mott–Littleton approach and QM/MM electronic-structure calculations. The predicted formation energies of oxygen vacancies accompanied by different excess electron localization patterns at the MM level of theory reach the accuracy of density functional theory (DFT) calculations using hybrid functionals. The new potential also accurately reproduces a wide range of physical properties of CeO_2_, showing excellent agreement with experimental and other computational studies. These findings provide opportunities for accurate large-scale modeling of the partial reduction and nonstoichiometry in CeO_2_, as well as a prototype for developing robust interatomic potentials for other defective crystals.

## 1 Introduction

Ceria (CeO_2_) is a technologically important rare-earth oxide with broad applications in several areas, including heterogeneous catalysis and solid oxide fuel cells (SOFCs).^1−3^ It is versatile in catalytic applications because of its unique defect properties. Apart from its critical role in automobile three-way catalysts, ceria also holds great promise in novel catalytic processes such as selective methane oxidation to methanol, CO_2_ conversion reactions, and hydrogen production through water splitting.^4−6^ CeO_2_ has outstanding oxygen storage capacity, which arises from its variable stoichiometry enabling the release or uptake of oxygen to pass through redox cycles.^7^ As a consequence, reduced ceria (CeO_2–*x*_) is widely used as a supporting material for single-atom/nanocluster catalysts with exceptional catalyst-stabilizing and oxygen-releasing capabilities.^8−10^ In addition, the high ionic conductivity of CeO_2_ is necessary for its use as an electrolyte in SOFCs and relies to a large extent on the rapid oxygen-vacancy formation and migration processes.^11,12^ Remarkably, CeO_2_ remains very stable in the cubic fluorite structure over a wide range of temperatures. The earth-abundant nature of cerium^13^ further makes it promising for large-scale industrial applications.

Oxygen vacancies are known to have low formation energies in ceria.^7^ Accompanied by the removal of an oxygen ion to form a doubly ionized vacancy V_O_^••^, two excess electrons could localize on two cation sites and form Ce_Ce_^′^ small polarons^14,15^ or be compensated by an oxygen interstitial O_i_^″^ to form an anion–Frenkel pair.^16^ Here, we employ the Kröger–Vink notation^17^ for a point defect M_S_^C^, in which M is the defect species, S is the lattice site that the defect occupies, and C is the charge of the defect relative to the original site (dots, primes, and crosses represent positive, negative, and neutral charges, respectively). For the former process, Paier et al.^7^ proposed that the best estimate of the charge-neutral oxygen vacancy formation energy in bulk ceria could be 4.2 ± 0.3 eV (relative to (1/2)E_O_2__) in O-rich conditions deduced from conductivity and calorimetric titration experiments, close to the heat of reduction from CeO_2_ to Ce_2_O_3_ of 4.0 eV.^18,19^ Surface vacancies have much lower (1–3 eV) formation energies than those in the bulk, depending on the facets and concentrations.^20−24^

In recent years, much effort has been devoted to studying the defect chemistry in CeO_2_ by plane-wave density functional theory (DFT) techniques using periodic supercell approaches.^7,25−31^ CeO_2_ is a strongly correlated oxide. The description of the highly localized 4*f* states becomes problematic in DFT calculations when Ce^4+^ is reduced to Ce^3+^. Standard Kohn–Sham DFT calculations using semilocal generalized gradient approximation (GGA) functionals such as Perdew–Burke–Ernzerhof (PBE) predict an unrealistic delocalized spin density for the occupied *f* states due to the self-interaction error (SIE).^25^ While the Hubbard U correction scheme^32^ (DFT+U, U_Ce4*f*_ = 4.5–6.0 eV) can solve the electron localization problem,^7^ the calculated formation energy of the oxygen vacancy (from 2.84 to 3.27 eV^26−29^) is underestimated by 1.0–1.5 eV compared with experiment (4.2 ± 0.3 eV^7^). Moreover, the choice of the U value is not straightforward, as it introduces uncertainty in the description of bulk properties, defect formation, and surface reactivity of ceria.^31,33^ It has been reported that with the increase in U the lattice constant of CeO_2_ becomes too large with increasingly underestimated oxygen vacancy formation energy.^31^ Furthermore, Branda et al.^34^ reported a variation of the oxidation state of Au on CeO_2_(111) with the U parameter. Watson et al.^25,26^ proposed employing the DFT+U correction also to the O_2*p*_ states, which further properly localizes holes in ceria trapped by native defects and impurities. However, this approach underestimates the formation energy of the oxygen vacancy even more: 2.22 eV based on U_Ce/O_ = 5.0/5.5 eV compared with 2.60 eV using U_Ce/O_ = 5.0/0 eV. Alternatively, hybrid functionals that include a fraction of the nonlocal Fock exchange, although computationally more demanding, yield results in much closer agreement with experiment. Several hybrid functionals have been used to study the bulk and defect properties of CeO_2_ based on periodic models.^30,31,35−37^ Notably, the oxygen vacancy formation energies predicted by hybrid functionals are much closer to experiment than the DFT+U predictions: 3.84 eV using PBE0^38^ and 3.63–4.09 eV using HSE06.^7,30,31^ Such improvements from hybrid functionals were also seen in other metal oxide systems with accurately predicted defect processes.^39^

The configurations of oxygen vacancies and the associated Ce_Ce_^′^ sites in reduced CeO_2_ have been a topic of considerable debate. Early theoretical studies assumed that the two excess electrons are trapped at the nearest neighbor (NN) sites of the vacancy (i.e., NN-NN).^20,25,40^ Later, DFT+U calculations performed by Wang et al.^41^ favored the localization of both electrons at the next-nearest neighbor (NNN) sites (i.e., NNN-NNN). In contrast, Murgida et al.^29^ obtained three configurations (one NN-NNN and two NNN-NNN configurations) sharing the same lowest formation energies. Despite the minor differences, these predictions are generally consistent. Early molecular-mechanical (MM) Mott–Littleton (M-L) calculations on doped ceria also suggested that large trivalent cations prefer to locate at the NNN sites over the NN sites.^42^ Scanning-tunneling microscopy (STM) imaging over the CeO_2_(111) surface conducted by Jerratsch et al.^43^ captured several different configurations, indicating that at least one Ce_Ce_^′^ is not adjacent to the vacancy site. The polarization energy of V_O_^••^ is large due to the high dielectric constants of CeO_2_, which can stabilize isolated charged species with respect to the neutral lattice, including both V_O_^••^ and Ce_Ce_^′^. Besides, the formation of Ce_Ce_^′^ requires an expansion of the surrounding lattice, which opposes the location next to the oxygen vacancy site where the neighboring Ce–O bonds shrink. As a result, the binding energy of V_O_^••^ with the NN Ce_Ce_^′^ polaron is very low (*ca*. 0.1 eV as reported by Sun et al.^30^), indicating that Ce_Ce_^′^ polarons are not tightly bound to oxygen vacancies. Experimentally, early conductivity measurements by Tuller and Nowick^44^ on single-crystal CeO_2–*x*_ samples also revealed that V_O_^••^ is the predominant charge state at a small *x* in CeO_2–*x*_ (*x* < 10^–3^), with a transition to singly ionized V_O_^•^ at a larger *x* in more reducing environments. In addition, a recent cathodoluminescence spectroscopy study by Thajudheen et al.^45^ demonstrated that the relative concentration of different charge states of oxygen vacancies depends on the oxygen partial pressure. Luo et al.^16^ observed using neutron scattering that the anion–Frenkel pair is dominant in the bulk, while Ce_Ce_^′^ polarons tend to aggregate at the surface and form an ordered reduced phase from nanorod samples. Such complexity in the charge compensation mechanism of oxygen vacancies could have a substantial impact on the ionic conductivity in SOFCs^12^ and surface reactivity when CeO_2_ serves as a catalyst^46^ or support.^47^ To resolve these uncertainties, reliable theoretical techniques are required to study the environmentally dependent formation mechanisms of all the relevant charge states of intrinsic defects in CeO_2_.

While being well-suited to modeling bulk materials and delocalized states, periodic boundary conditions have inherent limitations in modeling localized states such as isolated defects, polarons, and adsorbed molecules due to spurious image–image interactions. The long-range Coulomb interactions between periodic images originating from the net dipole moment of localized states are non-negligible.^48^ As a result, periodic DFT calculations on defective solids usually require a large supercell to mitigate these spurious periodic interactions,^7,29^ and additional correction schemes are usually necessary to achieve a higher level of accuracy.^48−50^ Also, for some highly charged defects, the finite-size supercells could still be insufficient to fully consider the long-range atomic perturbations. In particular, the formation of highly charged defects in CeO_2_, such as Ce_i_^••••^ and V_Ce_^⁗^, could provide a major perturbation of the surrounding atomic structure. These errors could be magnified when defect clusters are considered. Additionally, it is computationally very demanding with hybrid functionals to employ a sufficiently large supercell to minimize these errors.

A minor change in the formation energy of a point defect could result in several orders of magnitude variations in its calculated concentration, which could strongly affect the predicted electronic and optical properties.^39^ Therefore, minimizing errors in the modeling method is necessary to ensure the accuracy of defect predictions. Embedded-cluster techniques that naturally avoid periodic image interactions and further consider long-range polarization effects are an effective approach to modeling localized defects in solids at the dilute limit.^51−56^ The Molecular Mechanical, Mott–Littleton (MM M-L) approach^57−59^ and hybrid quantum mechanical/molecular mechanical (QM/MM) embedded-cluster techniques^55,60,61^ are widely used for modeling defect processes in solids. Both methods are based on the embedded-cluster framework: the QM/MM model includes a QM core, an interface, and part of MM atoms in the active region, while the M-L model only has one active MM region, which is allowed to relax fully in response to the formation of charged defects; the outer fixed part reproduces the infinite bulk environment.^59^ The M-L approach treats the outer part within a harmonic approximation to reproduce the bulk crystal field and linear dielectric response, while our QM/MM model implements a simplified continuous dielectric medium approximation where the response to the defect charge is calculated *a posteriori*.^55^ Furthermore, the shell-model^62^ interatomic potential (IP) is typically used in both techniques, in which the contributions of electronic long-range polarization effects due to the defect formation to the total energy can be computed through shell relaxations. With the rapid development of high-performance computing platforms, embedded-cluster calculations routinely employ over one thousand or more atoms in the active region (including QM and MM atoms) to consider explicitly the atomic displacements caused by defects, ensuring accuracy in predicting the formation energies and spectroscopic features.^54,63^

M-L calculations of defects in solids are performed at the pure MM level, which relies heavily on the accuracy of the IP. A reliable shell-model IP is a fundamental prerequisite for quantitively predicting the defect structures and formation energies. In contrast, embedded-cluster results commonly use DFT calculations employing hybrid functionals (although higher level theory may be used^64−66^) and with the IP-predicted dielectric response. Hence, an intrinsically consistent prediction of defect structures and formation energies by both approaches using the same potential is the ultimate goal that demonstrates the robustness of an IP. In previous work, such consistent predictions were achieved for MgO^67,68^ and ZnO^51,69^ but were limited to a few charge states of point defects without holes or electrons.

**Figure 1:**
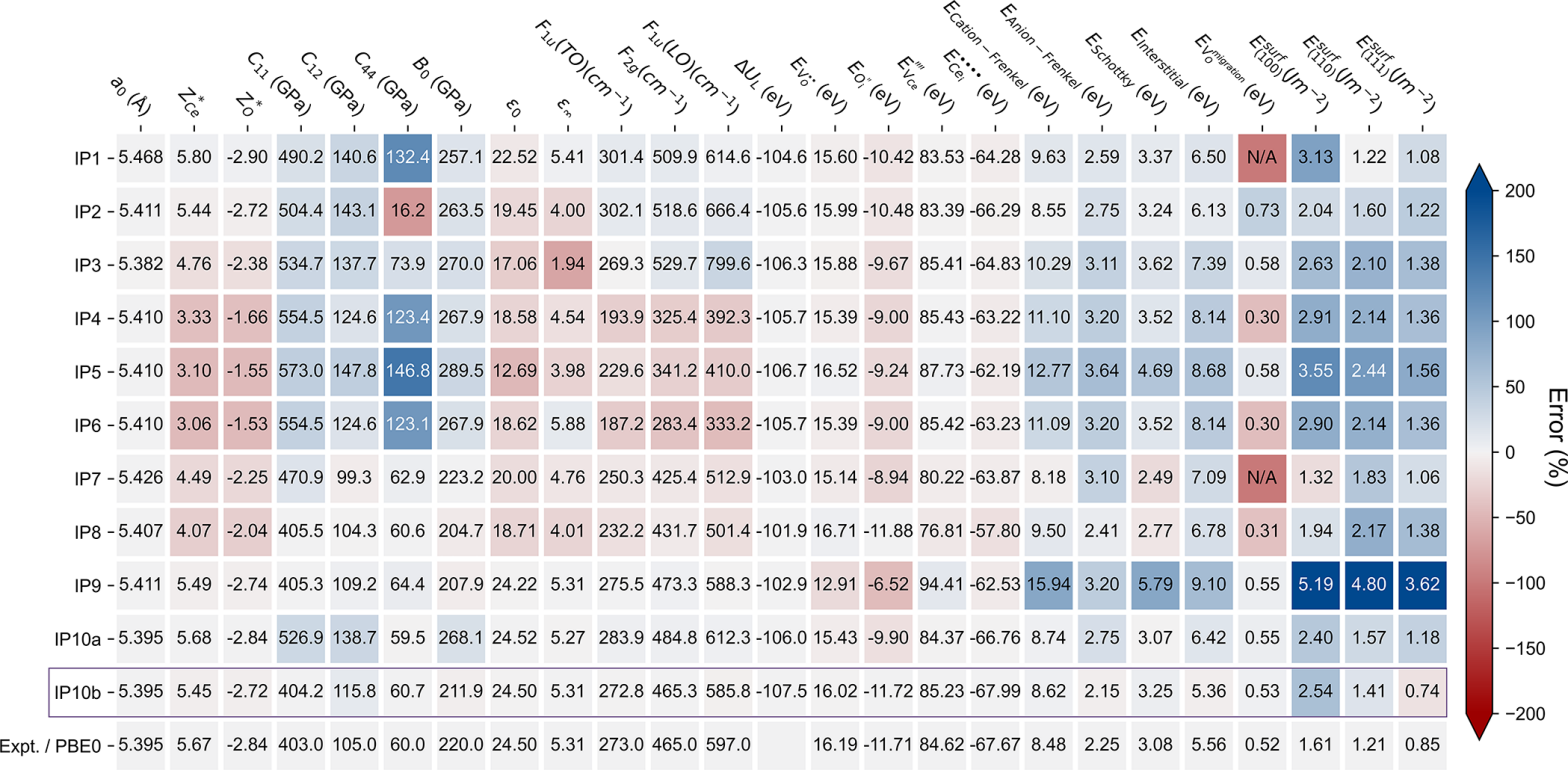
Performance of the previous (IP1-IP9) and our newly developed (*IP10a* and *IP10b*) shell-model potentials for modeling CeO_2_, compared with experimental and first-principles results. *IP10a* is developed based on the pure Buckingham potential in short-range interactions, while *IP10b* is the refined final version with improved performance, using a more complex potential form. The calculated values of each observable based on the corresponding potential are shown in the blocks. The blocks are colored according to the relative errors of the predicted properties compared with these experimental or theoretical references. Lattice constant *a*_0_ (extrapolated to 0 K),^33,81^ elastic constants *C*_11_, *C*_12_, and *C*_44_,^82^ phonon frequencies *F*_1u_(TO), *F*_2g_, and *F*_1u_(LO),^82^ static and high-frequency dielectric constants *ε*_0_ and *ε*_∞_,^83^ bulk modulus *B*_0_,^84^ and migration barrier of oxygen vacancy *E*_V_O__^migration^(85) are from previous experiments. References for Born effective charges Z_Ce_^*^ and Z_O_^*^ and surface energies *E*_(100)_^surf^, *E*_(110)_^surf^, and *E*_(111)_^surf^ are calculated at the PBE0 level of theory using VASP. References for the defect energies including *E*_V_O__^••^, *E*_O_i__^*″*^, *E*_V_Ce__^⁗^, and *E*_Ce_i__^••••^ and defect pair/trio formation energies *E*_cation–Frenkel_, *E*_anion–Frenkel_, *E*_Schottky_, and *E*_interstitial_ are calculated based on our hybrid QM/MM embedded-cluster model at the PBE0 level.

To date, a number of shell-model potentials have been developed for CeO_2_, which are collected in Table S1.^42,70−76^ IP-based computational studies of defect chemistry in ceria were first performed by Butler, Catlow, Cormack et al.^70,77,78^ using the M-L approach; these studies calculated vacancy migration energies in good agreement with experiment and demonstrated the crucial role of the ionic radius of dopants in determining the solution energy and the magnitude of dopant–vacancy interactions. Periodic models were also exploited to investigate the oxygen vacancy formation on surfaces and its role in CO oxidation.^71,79,80^ Despite these advances, no single IP can offer a consistently accurate description of all the physical and chemical properties of CeO_2_, as shown in Figure 1. It was possible to reproduce accurately the main bulk properties of ceria, including phonon frequencies and the oxygen vacancy migration barrier based on a more complex potential model (IP9 in Table S1),^76^ but the defect formation and surface energies proved to be significantly overestimated. One of the critical reasons for the less satisfying performance of previous potentials is the lack of reliable reference data in the IP development that exacerbates the errors in empirical parametrization.

In this work, we propose a novel methodology for IP development assisted by the QM/MM approach, which allows us to obtain accurate reference data for ionic polarizabilities, structure and formation energies of polarons, and intrinsic defects in dielectric materials. A new shell-model IP has been developed for CeO_2_ that reproduces accurately the experimental structure, elastic and dielectric constants, defect and surface properties, and phonon dispersion. This approach also provides references for parametrizing localized holes and polarons, endowing powerful capacities for modeling the localization of charge carriers accompanied by defect formation. Based on the new IP, the calculated structure and formation energies of charged defects by the M-L and QM/MM approaches using hybrid functionals achieve a very high level of consistency, which is greater than that achieved in previous work. Our potential is parametrized entirely using the shell-model and pairwise potentials without complex many-body interactions, ensuring excellent computational efficiencies in studying complex defective systems. Moreover, the newly proposed strategies for developing reliable shell-model potentials for accurate defect predictions assisted by QM/MM calculations could be extended to other systems.

## 2 Methodology

This work combines various computational techniques including plane-wave DFT calculations, IP-based lattice and M-L defect calculations, and the hybrid QM/MM embedded-cluster approach.

### 2.1 Plane-Wave DFT Calculations

Plane-wave DFT calculations were performed at several levels of theory using the Vienna Ab-initio Simulation Package (VASP)^86^ to set theoretical references for developing new IPs. Full computational details for calculating the bulk and surface properties of ceria are given in Section S1.1 of the Supporting Information.

### 2.2 Interatomic-Potential-Based Calculations

The development of IPs and IP-based calculations are performed based on the Born model of ionic solids^87^ using the General Utility Lattice Package (GULP) code.^88,89^ In traditional shell-model potentials, pairwise interactions are described by the Buckingham potential:
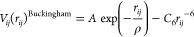
1where *r*_*ij*_ is the distance between two interacting ions and *A*, ρ, and *C*_6_ are the parameters. Ionic polarizability is treated by the shell model, in which the massless shell is connected to the atomic core by a harmonic spring with a spring constant *k*_2_:
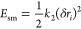
2The sum of the core and shell charges on an ion equals its formal charge. The ionic polarizability α in vacuum in the shell model is given by
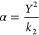
3where *Y* is the shell charge. The electrostatic Coulomb interaction is calculated as
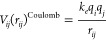
4where *k*_*e*_ is the dimensional Coulomb constant (14.3996 eV Å e^–2^).

In this work, our new potential keeps the classical Buckingham form for describing the O–O and Ce–Ce interactions, while taking a complex form combining repulsive and attractive terms for Ce–O interactions parametrized in GULP. This potential can be represented by using the Buckingham, 12-6 Lennard-Jones, Morse-like, and constant offset (0-order polynomial) analytical forms in GULP:
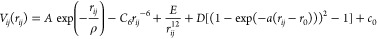
5where *A*, ρ, *C*_6_, *E*, *D*, *a*, *r*_0_, and *c*_0_ are the parameters of the potential.

IP-based defect calculations were undertaken based on the M-L approach.^57,58^ A point defect or defect cluster is embedded in the central part of the model (region I), where ionic relaxations and polarization are allowed explicitly. The surrounding regions (region IIa and IIb) that model the infinite solid are described within the linear response approximation. Region IIa acts as the interface, in which interactions with region I ions are calculated by explicit summation, but ionic displacements are calculated self-consistently using a harmonic approximation for the defect energy expanded around defect equilibrium configuration, as first proposed by Norgett^90^ and implemented in the GULP code.^88^ Region IIb extends to infinity to reproduce the periodic electrostatic environment of the solid, in which the long-range polarization energy of a charged defect outside the active region I is calculated using the macroscopic dielectric response of a perfect crystal (without a defect). The radii of region I and region IIa were set to 25 Å and 40 Å, respectively, which proved to be sufficiently large for considering the long-range polarization effects and provide well-converged defect energies (<0.1 eV).

Details of IP-based surface and vacancy migration calculations are described in Sections S1.2 and S1.3 of the Supporting Information.

### 2.3 QM/MM Calculations

We employ the hybrid QM/MM embedded-cluster approach to model defect formation in bulk CeO_2_ as implemented in the ChemShell^60,91^ code. Our hybrid QM/MM embedded-cluster model is divided into five regions. In the QM region, electronic-structure calculations are performed at the hybrid DFT level using NWChem.^92^ We have tested the performance of several pseudopotentials and basis sets to balance the computational accuracy and efficiency in large-scale QM/MM calculations of CeO_2_ (with 100–200 QM atoms). The combination of the Def2-TZVP basis set^93^ for O and a [4*s*4*p*2*d*3*f*] basis set developed by Erba et al.^94,95^ based on the Stuttgart–Dresden quasi-relativistic small-core (28 core electrons) effective core potential (ECP)^96^ for Ce was determined as the best choice. The differences in the calculated formation energies of defects and ionization potential between the current setting and more complete basis sets are less than 0.1 eV. We utilized three hybrid functionals for defect calculations: hybrid GGA functional B97-2^97^ and PBE0^98^ and hybrid meta-GGA functional BB1K,^99^ which include 21%, 25%, and 42% HF exact exchange, respectively. For consistency, we selected PBE0 results as the reference data in the IP development, together with those from VASP calculations using the same functional.

The MM calculations are performed using GULP. The MM part of the QM/MM structural model is divided into two regions: the MM-active region, which is allowed to relax during the calculations, and the MM-frozen region, which is fixed to reproduce the effect of the bulk environment. The outermost layer of the entire model includes point charges, which were fitted to eliminate the effects of surface termination and reproduce the Madelung potential of CeO_2_.

The interface region participates in both QM and MM interactions, serving as the buffer layer to minimize the mismatch of the QM and MM levels of theory. We used a specially designed local pseudopotential on the cationic sites in the form of a linear combination of three Gaussian functions.^56^ The fundamental idea and detailed procedures of the QM/MM interface treatment are shown in Section S1.4 of the Supporting Information. The parameters were trained with a least mean square procedure for residual gradients on the ions in the active (QM, interface, and MM-active) region and the scatter of deep core levels (in this study, O_1*s*_) in the Kohn–Sham spectrum. The fitted pseudopotential for the interface cations has the form of
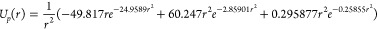
6

QM/MM defect calculations were performed with the python-based version of ChemShell (Py-ChemShell)^91^ on a large embedded-cluster model with ∼10 000 atoms in total and an O-centered 197-atom QM cluster focusing on the formation of oxygen vacancies. The accurately calculated energies of polaron formation at the NN and NNN sites have been used for validation and refinement of the interatomic potentials that we report. A Ce-centered 111 QM-atom model was used for other types of defects, which yields convergence of defect formation energies to *ca*. 0.1 eV.

### 2.4 Calculations of Defect Formation Energies

Lattice energy is the energy of the ionic compound with respect to constituent ions in the gas phase. The lattice energy Δ*U*_*L*_ can be calculated according to the Born–Haber cycle^100^ from experimental thermodynamical data, which (for CeO_2_) is given by
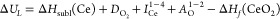
7where Δ*H*_subl_(Ce) is the sublimation enthalpy of Ce (4.380 eV^101^), *D*_O_2__ is the dissociation energy of O_2_ (5.136 eV^101^), *I*_Ce_^1–4^ is the sum of the first four ionization potentials of Ce (73.745 eV^101^), *A*_O_^1–2^ is the sum of the first (*A*_O_^1^) and second (*A*_O_^2^) electron affinities of O, and Δ*H*_*f*_(*CeO*_2_) is the formation enthalpy of CeO_2_ (−11.28 eV^101^). While *A*_O_^1^ is experimentally known as 1.461 eV,^102^*A*_O_^2^ is dependent on the atomic environment of oxides, adding uncertainty to the IP development and subsequent defect formation calculations. Freeman and Catlow suggested a value of 8.75 eV for SnO_2_, while Waddington^103^ obtained an average value of 9.41 eV from several oxides. Previous work has shown that Δ*U*_*L*_ is a critical quantity that strongly affects the accuracy of M-L defect calculations.^104^ However, fitting the potential to reproduce the “experimental” Δ*U*_*L*_ according to an arbitrary choice of *A*_O_^2^ may be problematic and could lead to several electronvolt errors in the calculated formation energies. We propose a self-consistent approach to determine the value of *A*_O_^2^ in CeO_2_ (8.14 eV, and therefore Δ*U*_*L*_ = −107.5 eV according to eq 7) based on QM/MM calculations of defect energies and plane-wave DFT calculations of surface energies, which will be discussed in the following sections. For the defect formation energy calculations using the Born–Haber cycle based on the M-L results, a consistent usage of the *A*_O_^2^ and Δ*U*_*L*_ predicted by the IP gives accurate results that are comparable with QM/MM calculations.

It is necessary to clarify the definition of some energy terms in defect calculations. In QM/MM calculations, the formation energy of a defect in the charge state of *q* is defined as
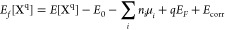
8where *E*[X^q^] and *E*_0_ are the calculated total QM/MM energies of the defect and perfect structures, *n*_*i*_ is the number of species that have been added (*n*_*i*_ > 0) or removed (*n*_*i*_ < 0) from the system to form the defect, *μ*_*i*_ is the chemical potential of the species *i*, and *E*_*F*_ is the Fermi energy relative to the valence band maximum (VBM). In order to account for the long-range polarization effect for charged defects that extends to infinity, an *a posteriori* correction is applied using the Jost formula,^61,105^
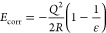
9where *R* is the radius of the active region (including QM, interface, and MM-active regions) and *Q* is the net charge of the defect system. High frequency *ε*_∞_ and static *ε*_0_ dielectric constants are used for vertical and adiabatic processes, respectively.

M-L calculations compute the defect energy *E*[X^q^] taking the gas phase ions (O^2–^(g) and Ce^4+^(g)) as the reference, which differs from the formation energy (with respect to O_2_(g) and Ce(s)) in conventional DFT calculations. The energy of O_2_(g) and Ce(s) is not defined in IP-based calculations. Instead, such energies can be in turn obtained from the Born–Haber cycle^100^ and relate *E*[X^q^] to *E*_*f*_[X^q^] by
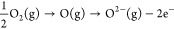
10
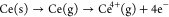
11Therefore, the energy of the added or subtracted species in defect formation can be obtained by
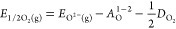
12
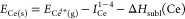
13Accordingly, the defect energies *E*_V_O__^••^, *E*_O_i__^″^, *E*_V_Ce__^⁗^, and *E*_Ce_i__^••••^ can be directly obtained and compared in M-L and QM/MM approaches. Furthermore, the energy changes during the formation of localized electrons or holes trapped by the defects do not include the fourth ionization potential of Ce (*I*_Ce_^4^, 36.762 eV^101^) or *A*_O_^2^, which should be subtracted from the M-L-calculated defect energies.^100^ Defect energies calculated by the M-L approach implemented in GULP have already accounted for the long-range polarization effect, so extra corrections are not needed.

For example, when an oxygen vacancy V_O_^*q*^ with a charge state *q* (*q* = 0, +1,+2) is formed in CeO_2_, the excess 2 – *q* electrons are not trapped at the vacancy site but will localize on the nearby Ce site to form Ce_Ce_^′^ small polarons. This process can be formed thus:
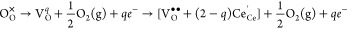
14The formation energy determined using M-L calculations is then calculated as the corresponding reaction energy:
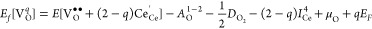
15where *E*[V_O_^*q*^] = *E*[V_O_^••^ + (2 – *q*)Ce_Ce_^′^] is the defect energy obtained from an M-L calculation and *E*_*F*_ is the Fermi level relative to VBM. Similarly, a cerium vacancy with a charge state *q* (*q* = 0, −1, −2, −3, −4) can trap (4 + *q*) localized holes O_O_^*•*^. The formation energy of V_Ce_^*q*^ can be calculated by
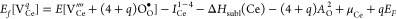
16Also, the formation energies of oxygen interstitials *E*_*f*_[*O*_*i*_^*q*^] are calculated by
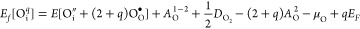
17Finally, the formation energies of cerium interstitials *E*_*f*_[*Ce*_*i*_^*q*^] are calculated by
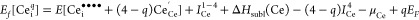
18

The formation energy of a charged defect is also dependent on the growth conditions. In the O-rich/Ce-poor conditions, the upper limit is determined by the formation of O_2_ molecules, in which μ_O_ = 0 eV and μ_Ce_ = Δ*H*_*f*_(CeO_2_). In O-poor/Ce-rich conditions, the lower limit of μ_O_ is determined not only by CeO_2_ but also by other Ce*_n_*O*_m_* phases, where CeO_2_ should remain more stable than any other reduced phases. Conventionally, we take Ce_2_O_3_ as the limiting phase as employed in previous DFT calculations.^27,28,30^ Hence,
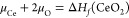
19
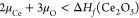
20Therefore, the chemical potentials should satisfy
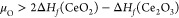
21
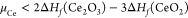
22Thermochemical experimental measurements give Δ*H*_*f*_(CeO_2_) = −11.28 eV and Δ*H*_*f*_(Ce_2_O_3_) = −18.62 eV,^101^ which allows us to calculate μ_O min_ = −3.94 eV and μ_Ce max_ = −3.40 eV, which were used in our calculations. In QM/MM calculations, the reference energies for an O_2_ molecule and a single Ce^4+^ ion are calculated using NWChem with the corresponding basis set and functional. The sum of the first four ionization potentials of Ce (*I*_Ce_^1–4^, 73.745 eV^101^) and sublimation enthalpy of Ce (Δ*H*_subl_(Ce), 4.380 eV^101^) are then used to obtain the reference energy of Ce(s).

In both M-L and QM/MM calculations, formation energies (per defect) of the unbound cation–Frenkel pair, anion–Frenkel pair, Schottky trio, and interstitial disorder defects were calculated by
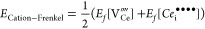
23
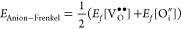
24
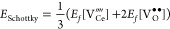
25
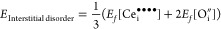
26

## 3 Results and Discussions

### 3.1 Strategies for Developing Robust Shell-Model Potentials

In this work, we report a framework for developing a robust shell-model IP for CeO_2_ assisted by QM/MM calculations, which could also be extended to other ionic solids. First, a classical shell-model IP based on Buckingham potentials was parametrized to reproduce the bulk structure and dielectric constants. As discussed in the previous section, to make the optimal choice, we systematically reviewed the performance of several candidate potential parameter sets based on different values of the ρ parameter in modeling defects and surface properties. Next, the optimal potential, *IP10a*, was used in hybrid QM/MM embedded-cluster models to obtain accurate references of the structure and formation energies of intrinsic defects in CeO_2_, including oxygen vacancies, cerium vacancies, oxygen interstitials, cerium interstitials, hole polaron, and electron polaron. Then, these data were used in turn to optimize the Ce^4+^–O^2–^ interactions using a more complex form that combines the Buckingham, Lennard-Jones, and Morse potentials for correcting the derivatives and an offset constant for correcting the lattice energy, *IP10b*. Finally, the Ce^3+^–O^2–^ and Ce^4+^–O^1–^ potentials were fitted based on the Ce^4+^–O^2–^ potential according to QM/MM calculations of electron and hole polarons in terms of bond length and formation energy. Based on the newly developed *IP10b*, the calculated defect structures and formation energies at the MM level of theory using the M-L approach are highly consistent with QM/MM results using hybrid DFT functionals.

#### 3.1.1 Construction of the Reference Data Set

CeO_2_ is a widely studied material with well-documented experimental measurements of physical and chemical properties. However, due to the easily reducible nature, oxygen vacancies and electron polarons in CeO_2_ could significantly affect the measurements, resulting in inconsistent reports of several properties. For example, a considerable concentration of Ce^3+^ (12%–45%) was detected in many experimentally synthesized polycrystalline thin-film samples, which are very difficult to eliminate even with oxygen plasma treatment at high temperatures.^106,107^ Such a high concentration of Ce^3+^ in CeO_2_ could originate not only from surface defects but also from amorphous Ce_2_O_3_ formed at the grain boundaries of nanocrystals.^106^ Hence, we focus on data obtained from single crystal and stoichiometric samples if available. The experimental lattice constant *a*_0_ of undoped CeO_2_ is typically reported to be 5.411 Å at room temperature.^108−110^ Gupta and Singh reported an *a*_0_ of 5.401 Å at 100 K, and no data are available close to 0 K. Based on several thermal expansion experiments, we obtained an estimate for *a*_0_ of 5.395 Å when extrapolated to 0 K.^81,111−113^ This estimate also allows us to obtain an *a*_0_ of 5.411 Å at 300 K based on the new IP using free energy calculations within the quasi-harmonic approximation (20 × 20 × 20 *k*-points on the 12-atom conventional cell of CeO_2_), in excellent agreement with experiment.

The O_2*p*_ → Ce_4*f*_ band gap of CeO_2_ is typically reported ranging from 3.0 to 3.5 eV^3,106,114−116^ and is known to decrease with the increase in the concentrations of oxygen vacancies and Ce^3+^.^3,106^ However, Pelli Cresi et al.^117^ recently proposed a revised value of 4 eV for the optical band gap based on steady-state and ultrafast transient absorption spectra measured on stoichiometric samples. They argued that the long absorption tail from 3 to 4 eV in the Tauc plot should be identified as the Urbach tail, further confirmed by the photobleaching in the transient absorbance measurement, which corresponds to the occupied Ce 4*f* localized state due to the rapid small polaron formation. Such an Urbach tail is also seen in nanoparticle samples with a larger band gap of 4.2–4.3 eV due to the quantum confinement effect.^118^ This conclusion is also supported by previous high-resolution electron energy loss spectroscopy (EELS) measurements on stoichiometric^119^ and fully oxidized single-crystal^120^ samples, which located the unoccupied Ce_4*f*_ state 4–4.4 eV higher than the O_2*p*_ state. Hence, we consider that a band gap of 4 eV measured by Pelli Cresi et al.^117^ could be the best estimate for CeO_2_ and is further employed in this work. We will compare with experimental data for the elastic constants *C*_11_, *C*_12_, and *C*_44;_(82) zone-center phonon frequencies *F*_1u_(TO), *F*_2g_, and *F*_1u_(LO);^82^ static and high-frequency dielectric constants, *ε*_0_ and *ε*_∞_;^83^ bulk modulus *B*_0_;^84^ and migration barrier of oxygen vacancy *E*_V_O__^migration^.^85^

Plane-wave DFT calculations using VASP at different levels of theory were performed alongside our semiclassical GULP simulations. The calculated lattice constants, band gaps, Born effective charges, and surface energies of CeO_2_ are listed in Table 1, where we find that the hybrid functionals in our assessment set show a superior agreement with experiment. Previous work also showed that in general hybrid functionals perform much better than GGAs in describing the formation enthalpy, heat of reduction, and defect formation energy of CeO_2_.^7,30,36^ Because the PBE0 functional is implemented in both the plane-wave DFT code (VASP) and atomic-centered basis set code (NWChem), employed in our hybrid QM/MM calculations, we consistently use PBE0 results as the primary reference in the IP development. The Born effective charges and surface energies from VASP calculations, as well as the in-lattice ionic polarizabilities, defect structures, and energies from QM/MM results, were used in refining the IP. Our new IP can easily be reparameterized to reproduce the predictions from any other functional based on the same protocol.

**Table 1:** Calculated Lattice Constant *a*_0_, Band Gap *E*_g_, High-Frequency Dielectric Constant *ε*_∞_, Born Effective Charges Z_Ce_^*^ and Z_O_^*^, and Surface Energies of CeO_2_ Using VASP Compared with Previous Experimental and Theoretical Results

	*a*_0_ (Å)	*E*_g_ (eV)	*ε*_∞_	Z_Ce_^*^	Z_O_^*^	*E*_(100)_^surf^(J m^–2^)	*E*_(110)_^surf^(J m^–2^)	*E*_(111)_^surf^(J m^–2^)
PBE+U (U_Ce 4*f*_ = 5 eV)^a^	5.490	2.29	6.59	5.53	–2.77	1.45	1.06	0.69
PBEsol+U (U_Ce 4*f*_ = 5 eV)^a^	5.435	2.41	6.57	5.51	–2.75	1.77	1.27	0.90
PBE0^a^	5.397	4.38	5.67	5.67	–2.84	1.61	1.21	0.85
PBEsol0^a^	5.361	4.46	5.68	5.68	–2.84	1.83	1.33	0.96
HSE06^a^	5.396	3.63	5.71	5.67	–2.84	1.59	1.20	0.84
PBE+U (U_Ce 4*f*_ = 5 eV)^b^	5.489					1.44	1.06	0.71
PBEsol+U (U_Ce 4*f*_ = 5 eV)^c^	5.431		6.55	5.53	–2.76			
PBE0^d^	5.403	4.35						
PBE0^e^	5.401					1.64	1.27	0.86
PBEsol0^d^	5.368	4.40						
HSE06^f^	5.40	3.50						
GW_0_^g^		3.88						
Experiment	5.411^h^	2.9–3.3^k^	5.31^q^			1.20 ± 0.2 (averaged)^r^		
	5.401^i^	4.0^l^						
	5.395^j^	3.6–4.11^m^						
		4.0^n^						
		4.4^o^						
		4.3–4.4^p^						

a Present work.

b Reference (121).

c Reference (122).

d Reference (37).

e Reference (38).

f Reference (35).

g Reference (123).

h Room-temperature measurements.^108−110^

i Low-temperature measurement at 100 K.^113^

j Extrapolated to 0 K from thermal expansion measurements.^81,111−113^

k Spectroscopic ellipsometry measurement on nanocrystalline or single-crystal CeO_2–*x*_ films.^106,116^

l Steady-state and ultrafast transient absorption spectra measurement on stoichiometric CeO_2_ thin film. A revised optical band gap of 4 eV was proposed for bulk ceria, and the absorption tail below 4 eV was identified as the Urbach tail.^117^

m Optical measurement on polycrystalline CeO_2_ films with 140–180 nm thicknesses.^124^

n The electron energy loss spectroscopy (EELS) spectrum captured the O_2*p*_ → Ce_4*f*_ transition starting at 4 eV on single-crystal thin films, and the intensity between 0 and 3 eV indicates the Ce^3+^ state.^120^

o High-resolution EELS study on stoichiometric thin films observed a 4.4 eV energy loss from O_2*p*_ to the empty Ce_4*f*_ state transition.^119^

p Measurement from the optical absorption spectrum on the CeO_2_ nanoparticles.^118^

q Transmissivity and reflectivity measurement on CeO_2_ quasi-single crystal sample.^83^

r Measurement on nanoparticle samples by calorimetry methods.^125,126^

#### 3.1.2 Strategies for Improving the Accuracy of Shell-Model Potentials

A shell-model potential can be divided into two parts: the shell model (*Y* and *k*_2_) and other parameters including short-range repulsion and dispersion interactions. The shell-model defines the ionic polarizabilities that significantly affect the dielectric properties of materials, which play a key role in defect formation. Previous parametrizations of interatomic potentials for ceria have in common a somewhat unsatisfactory approach to choosing how the lattice polarizability is distributed between individual ions, which may not correctly reflect the physical nature of the target material. Polarizabilities have often been determined by fitting spring constants and shell charges to experimental data without consideration of the relative polarizabilities of cations and anions; it is possible to obtain a number of candidate potential sets with different shell-model parameters but with similar performances in modeling the bulk properties. An alternative to purely empirical fitting was developed by Lewis and Catlow^74^ considering in-lattice polarizabilities based on gas-phase Pauling’s polarizabilities of cations, which are known to be less affected by the crystal environment. By contrast, the anion polarizability varies strongly with the oxide structure and chemical composition.^74^ The in-crystal ionic polarizabilities can be calculated from hybrid QM/MM embedded-cluster models (*cf*. ref (127)). Here, we have employed ChemShell with NWChem as a QM driver to construct suitable embedded-cluster models and calculated the in-lattice ionic polarizability. The detailed methodology is presented in Section S1.5 of the Supporting Information.

In Table 2, we collected the calculated in-lattice polarizabilities of Ce^4+^ and O^2–^ ions using the Def2-TZVP^93^ basis set. It can be seen that the gas-phase cation polarizability is very close to the frozen in-crystal polarizability, as has been pointed out by Lewis and Catlow.^74^ The gas-phase calculations for O^2–^ are extremely basis-set-dependent, (as expected, since the O^2–^ ion is an unbound species in the gas phase), and increases from 6.293 Bohr^3^ using the Def2-TZVP basis set to 50.871 Bohr^3^ using Def2-QZVPPD^128^ at the PBE0 level of theory. In the limit of a complete basis set, the ground-state solution would include an electron dissociated from the oxide ion to infinity and, therefore, an infinite polarizability. The hybrid DFT predictions are in good agreement with higher-level coupled-cluster results. The intrinsic polarizability of an ion, *α*_*i*_, in the CeO_2_ crystal environment does not include effects of charge transfer from or to surrounding ions, which plays a significant role in real materials, and the total polarizability of an ion is
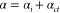
27where *α*_*ct*_ is the respective contribution from charge transfer. When fitting IP parameters, we kept the ratio of Ce^4+^ and O^2–^ polarizabilities (α = *Y*^2^/*k*_2_) constant according to the QM/MM calculated polarizability data, based on a conjecture that *α*_*ct*_ is proportional to *α*_*i*_. Under this methodology, a unique set of shell-model parameters can be determined that models more accurately the relative ionic polarizabilities in solids. Our results show that Ce^4+^ and O^2–^ have similar polarizabilities in CeO_2_. By calculating the ionic polarizabilities through eq 3, we found that most previous IPs cannot reproduce this feature.

**Table 2:** Calculated Intrinsic In-Lattice Ionic Polarizabilities (Bohr^3^) of CeO_2_ Using the ChemShell Embedded-Cluster Model Compared with Their Counterparts Calculated in the Gas Phase Where Possible (O^2–^ Ions Are Unstable in the Gas Phase)

	B97-2	PBE0	BB1K	HF	CCSD	CCSD(T)
*α*_*i*_ (O^2–^ in CeO_2_)	5.601	5.601	5.535	5.400	5.380	5.373
*α*_*i*_ (Ce^4+^ in CeO_2_)	5.901	5.935	5.894	5.915	5.886	5.900
*α*_*i*_ (gas-phase Ce^4+^)	5.842	5.871	5.832	5.852	5.825	5.836

Polarizability also determines the dispersion (London) interaction, the strength of which is usually described by the *C*_6_ coefficient in the Buckingham interatomic potential. The similarly polarizable Ce^4+^ and O^2–^ in CeO_2_ suggest that the *C*_6_ coefficients for Ce^4+^–O^2–^, Ce^4+^–Ce^4+^, and O^2–^–O^2–^ should all be considered, whereas most of the previous IPs did not introduce the *C*_6_ coefficients for all these interactions. We have calculated *C*_6_ according to the Slater–Kirkwood formula^129^ using the participation numbers reported by Pyper et al.^130^ and our calculated polarizability data. *C*_6_(XY) is given as
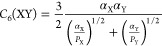
28where *P*_X_ is the participation number of ion X (4.455 for O^2–^ and 7.901 for Ce^4+^ from Pyper et al.^130^).

In ionic solids, the long-range electrostatic interaction contributes greatly (over 90%) to the total lattice energy. Although the short-range repulsive interaction originating from the overlapping electron densities contributes much less, the pairwise parameters in an IP largely control the bond lengths and structures and, hence, energies and physical properties. Among all the short-range interactions, the cation–anion interaction has the greatest effects due to its directly bonded nature. The cation–cation and anion–anion interactions are much weaker because of the longer interatomic distances. We note that parameter ρ in the previous Ce^4+^–O^2–^ Buckingham potentials varied from 0.335 Å to 0.429 Å. A small ρ with a large *A* or a large ρ with a small *A* may show only minor differences in the calculated bulk properties. However, we found that the choice of ρ is of vital importance in predicting defect and surface properties, which are typically not included in the list of fitted observables. Hence, the “best” IP derived by the fitting procedure may not be appropriate for modeling defects and surfaces, as can be seen in the predicted incorrect relative stabilities of surfaces and defect pairs by several IPs.

To explore the effects of ρ, we parametrized several IPs based on different fixed values of ρ within the traditional Buckingham potential framework (shown in Table S2). These potentials share the same Ce^4+^–Ce^4+^ and O^2–^–O^2–^ parameters, while other parameters are fitted to minimize the sum of squares of selected observables. We used the *A* and ρ parameters of the O^2–^–O^2–^ Buckingham potential from Lewis and Catlow^74^ since they have remarkable transferability in many ionic oxide systems and can be combined with other potentials to study dopants and solid solutions. We also put the highest fitting weights on the lattice constant (*ca*. 5.395 Å at 0 K) and static and high-frequency dielectric constants due to their critical importance in determining the accuracy of QM/MM calculations. Therefore, all the IPs we derived give accurate reproductions of the lattice constant and dielectric constants.

Figure S1 shows how the ρ parameter affects the predicted properties of CeO_2_. The dark gray horizontal lines represent the reference data from experiment or DFT calculations at the PBE0 level of theory. If we only consider the bulk properties that are normally used as fitting observables, a larger value of ρ, such as ρ = 0.37 Å, could be the result obtained by the least-squares procedure. However, if we further consider the defect and surface properties, those IPs with a larger value of ρ produce inaccurate results for ceria. By systematically considering bulk, defect, and surface properties (discussed in Section S1.6 of the Supporting Information), we determined the IP with ρ = 0.349 Å as the best Buckingham potential for CeO_2_, which was further used in QM/MM defect calculations. We stress that the QM/MM calculations based on *IP10a* are reliable and consistent with those based on *IP10b* since the lattice constants, dielectric constants, and ionic polarizabilities predicted by the two potentials are identical. The full set of parameters is provided in Table S2, and the performance in reproducing the properties of ceria has also been shown in Figure 1, denoted as “*IP10a*”. Within the current model, it is still impossible to reproduce simultaneously all targeted properties because the cation–anion short-range interaction cannot be accurately described by a single Buckingham potential. However, we have shown here that the shell-model potential with the classical Buckingham form can qualitatively and semiquantitatively reproduce the correct atomic structure and properties of CeO_2_ based on a careful selection of some critical parameters.

#### 3.1.3 Refining the Interatomic Potential According to QM/MM Calculations

Short-range interactions in solids sometimes cannot be exactly described by a single potential adopting a simple analytical form. The Lennard-Jones potential is known to be considerably too repulsive at short bond distances.^131^ Although the Buckingham (and its constituent Born–Mayer) potential is a good physically justified approximation for the short-range repulsion,^132^ empirical fitting procedures could cause unwanted problems, as illustrated by the critical choice of ρ described above. Combining different forms, nevertheless, could increase the parametric space and overcome the weakness of each component to improve the accuracy.^104,133^ Derivatives of an IP determine the structure and elastic, dielectric, and phonon properties of the modeling system. The absolute potential value determines the lattice energy, which further affects the calculated energies of defects and surfaces. To obtain a robust IP, both derivatives and absolute values of the potential should be accurately determined.

We define a new analytic form for the cation–anion pairwise potential, as shown in eq 5. Parameters of the refined potential (*IP10b*) for CeO_2_ based on this form are shown in Table 3. The sole Buckingham potential representing the short-range interaction is insufficient for a consistently accurate description of elastic, dielectric, and phonon properties. This weakness can be attributed to inaccurate potential derivatives near the first and second neighbor bond distances. Hence, a Morse-like function is superimposed on the Buckingham potential to correct these derivatives and reproduce these bulk properties accurately. The offset constant *c*_0_ shifts down the potential curve by a constant to correct the lattice energy. Weak Lennard-Jones potentials were used to add repulsion to the interactions at very short bond distances to overcome the Coulomb catastrophe that could occur in molecular dynamics or Monte Carlo simulations.^133^ The overall potential is truncated at 5.278 Å where the potential energy drops to 0 eV, between the second and third Ce–O neighbor bond lengths. The resulting potential curve is shown in Figure S2, compared with previous potentials in the literature. The shell model parameters were slightly optimized for better overall performance, while the ratio of polarizabilities of Ce^4+^ and O^2–^ remained consistent with *IP10a*.

**Table 3:** Optimized Potential *IP10b* (Defined in Equation 5) for CeO_2_ Combined with Parameters for Modeling Hole (O^1–^) and Electron (Ce^3+^) Polarons^a^

(a) Short-Range Potential										
Interaction	*A* (eV)	ρ (Å)	*C*_6_(eV Å^6^)	*E*(eV Å^12^)	*D* (eV)	*a* (Å^–1^)	*r*_0_ (Å)	*c*_0_ (eV)	*r*_min_ (Å)	*r*_max_ (Å)
O^2–^–Ce^4+^	1138.963021	0.417578	25.082349	1.0	–1.15172262	0.4	4.53327	–1.077767	0	5.278
O^2–^–Ce^3+^	1025.066719	0.417578	25.082349	1.0	–1.036550358	0.4	4.53327	–0.9249903	0	5.532
O^1–^–Ce^4+^	706.157073	0.417578	25.082349	1.0	–0.714068	0.4	4.53327	–0.7462155	0	5.278
O^2–^–O^2–^	22764.3	0.149	20.983768	1.0					0	15.0
O^1–^–O^2–^	22764.3	0.149	20.983768	1.0					0	15.0
O^1–^–O^1–^	22764.3	0.149	20.983768	1.0					0	15.0
Ce^4+^–Ce^4+^	1.0	0.1	30.481293	1.0					0	15.0
Ce^3+^–Ce^4+^	1.0	0.1	30.481293	1.0					0	15.0
Ce^3+^–Ce^3+^	1.0	0.1	30.481293	1.0					0	15.0

(b) Shell Model		
Species	*Y* (e)	*k*_2_(eV Å^–2^)
Ce^4+^ shell	13.85	1071.1845
Ce^3+^ shell	12.85	1071.1845
O^2–^ shell	–2.936345	53.022513
O^1–^ shell	–1.936345	53.022513

a Short-range potentials were designed only for shell–shell interactions. A GULP-readable format is also provided in Section S3 of the Supporting Information.

**Figure 2:**
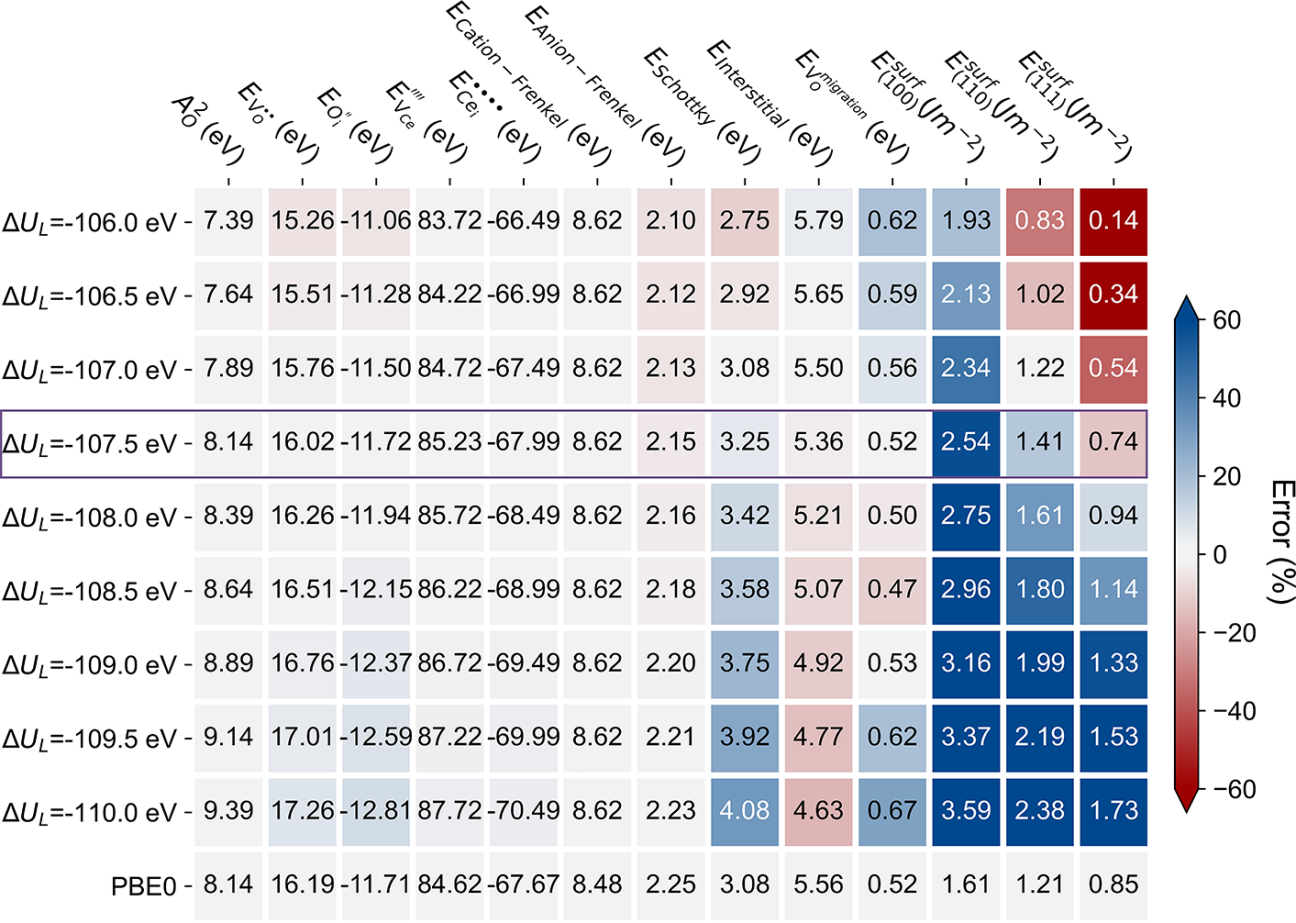
Effects of the predicted lattice energy Δ*U*_*L*_ on the calculated defect and surface properties of CeO_2_ based on the new potential formula.

We here also emphasize the critical role of the lattice energy predicted by a shell-model potential in determining the accuracy of defect and surface calculations. The lattice energy Δ*U*_*L*_ sets an essential basis for every calculation regarding energy differences such as surface energy, defect formation energy, and migration barrier. Lattice energy is not a direct experimental observable but can be evaluated theoretically through the Born–Haber cycle based on the experimental formation enthalpy, as shown in eq 7. However, the exact value of Δ*U*_*L*_ requires the knowledge of the oxygen second electron affinity *A*_O_^2^, which is a crystal-structure-dependent variable^134^ and not known from experiment. We proposed that the required Δ*U*_*L*_ and *A*_O_^2^ in the IP model can be determined according to DFT-calculated surface and defect energies. As shown in Figure 2, several variants of potentials are derived based on different Δ*U*_*L*_ values by only varying the *c*_0_ term while keeping all other parameters constant. As a result, these potentials share the same shape (derivatives) but differ in the absolute potential energy, thus predicting the same equilibrium-state structure but different absolute energies for defects and surfaces. The predicted defect and surface energies change considerably despite the minor differences in the predicted Δ*U*_*L*_. Periodic DFT calculations of surface energies and QM/MM calculations of defect energies at the PBE0 level were employed as references to obtain the required lattice energy that gives the most accurate prediction. In IP-based surface calculations, the lattice energy mainly determines the required energy to generate the unrelaxed surfaces rather than the relaxation energy. The most stable (111) surface has only 0.01 (0.05) J m^–2^ relaxation energy as predicted by periodic DFT (IP-based) calculations, which could be the best reference for the target lattice energy. We found that both defect and surface calculations support that Δ*U*_*L*_ = −107.5 eV, corresponding to an *A*_O_^2^ of 8.14 eV, which is employed as the final version of our CeO_2_ potential (*IP10b*). We note that the new potential still has certain errors on the calculated surface energies compared with periodic DFT results. To our knowledge, the only experimental measurement of surface energies of CeO_2_ was based on nanoparticle samples (which should be dominated by the most stable (111) surface) that reported an average value of 1.20 ± 0.2 J m^–2^,^125,126^ slightly higher than DFT predictions. The overestimation mainly originates from the cleavage energy before structural relaxation, which is also seen in other IP models. We have checked that the optimized surface structures are consistent with PBE0 predictions with an average error of 0.012 Å for the predicted surface and subsurface Ce–O bond lengths, suggesting a reasonably good description by the IP model.

#### 3.1.4 Parameterization for Hole and Electron Polarons in CeO_2_

Finally, we developed additional parameters for modeling localized electrons (Ce_Ce_^′^) and holes (O_O_^•^) in CeO_2_. The electron small polaron has been experimentally observed by STM imaging and confirmed by DFT calculations on the (111) surface.^12,30,40,43^ Holes also stabilize as small polarons in CeO_2_,^25^ which is a common feature of many oxide materials.^135^ The formation of electrons and holes could be modeled through the M-L approach as described by Freeman and Catlow in SnO_2_.^100^ Conventionally, electrons/holes were modeled by adding/subtracting a unit charge from the original shell charge and keeping the same core charge. However, using the same short-range potential to describe interactions between ions in different oxidation states is less accurate and usually results in several electronvolt errors in the predicted ionization potential, electron affinity, and band gap compared with experiment or DFT.^100,104^ Instead, we propose a correction scheme based on the new O^2–^–Ce^4+^ potentials but where we target the bond lengths and formation energies of small polarons obtained from our QM/MM calculations.

In both M-L and QM/MM calculations, vertical processes were modeled through electronic (shell) relaxation, while adiabatic processes required explicit structural optimization of the active regions. We performed QM/MM calculations to obtain the vertical and adiabatic ionization potentials and electron affinities. In the adiabatic processes, electrons and holes are self-trapped, forming small polarons in CeO_2_. Before fitting the potentials for modeling the polarons, the shell charges were first corrected in line with the formal charges of Ce^3+^ and O^1–^. Then, the coefficients in the Ce^4+^–O^2–^ potentials (*A* and *D*) are multiplied by a factor to correct the derivatives of the potential and reproduce the polaron structures. This factor was determined by targeting the average Ce^3+^–O^2–^/O^1–^–Ce^4+^ first-neighbor bond length around the ionized species calculated by the M-L approach to correspond to PBE0 QM/MM results. Finally, the potential is offset by a *c*_0_ constant to reproduce the polaron formation energy calculated by QM/MM. A similar procedure was used to parametrize the Ce^4+^–O^1–^ potential for describing localized holes, while the vertical ionization potential was used as the reference for correcting *c*_0_, ensuring the alignment of the positions of VBM in both techniques for accurate formation energy calculations of charged defects. Full details of the additional parameters have been given in Table 3.

In summary, we have provided some new strategies to develop shell-model potentials with improved performance in modeling ionic materials in Section 3.1. Implementation of in-lattice ionic polarizabilities, lattice energy, defect energies, and surface energies in the potential fitting improves the description of various physical properties, while consideration of QM/MM results of charge carriers further makes it possible to model various charged defects at the MM level of theory. Based on this approach, the formation of native defects in different charge states can be reasonably described through the M-L approach, assuming that the charge carrier is well localized and trapped by defects, as is the case in CeO_2_. Such an approximation gives reasonable accuracy with results comparable with DFT calculations using hybrid functionals, as will be shown in Section 3.2.

### 3.2 Performance of the New Potential in Modeling Defects in CeO_2_

**Figure 3:**
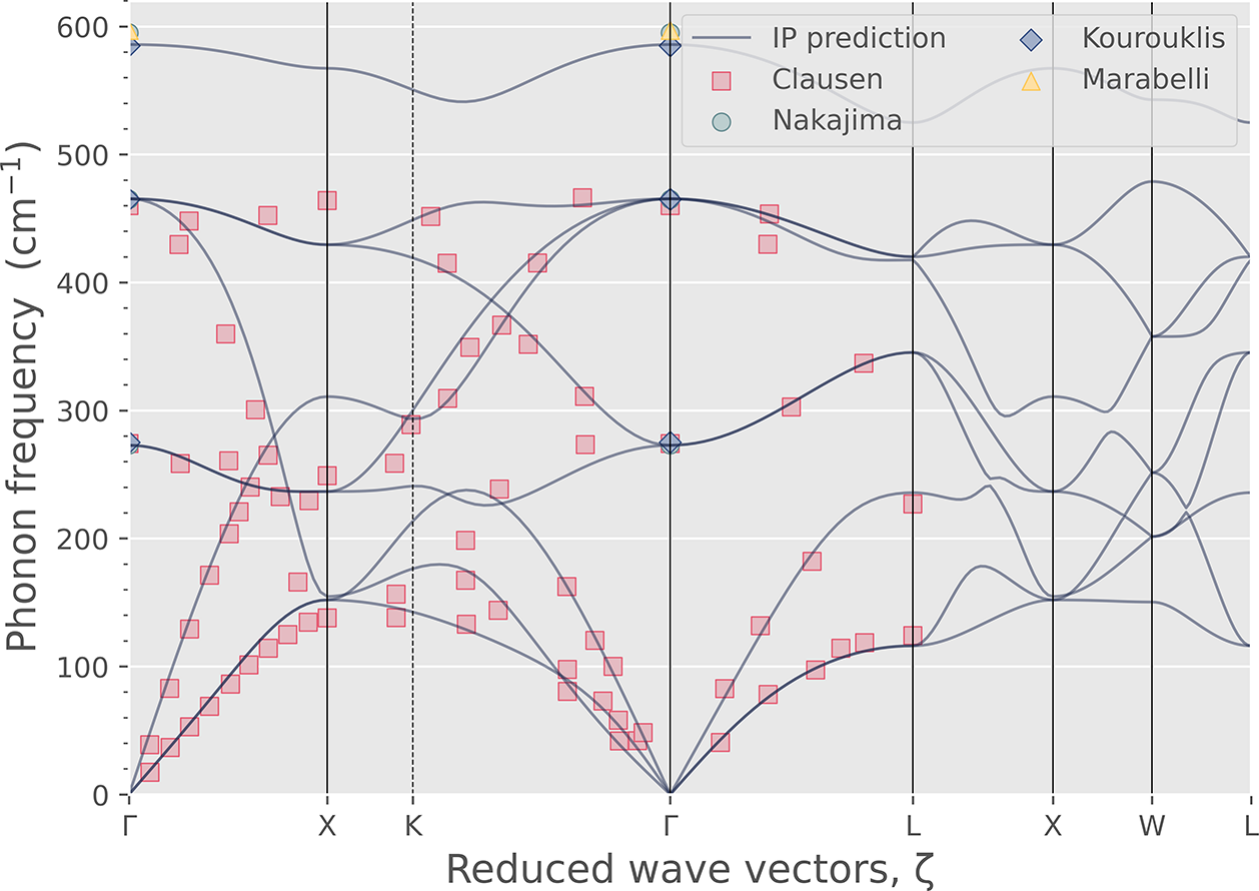
Calculated phonon dispersion for CeO_2_ based on the new potential compared with experimental data from Clausen et al.,^136^ Marabelli and Wachter,^137^ Nakajima et al.,^82^ and Kourouklis et al.^138^

The structure and properties of CeO_2_ predicted by the revised potential have been shown in Figure 1, denoted as *IP10b*. Compared with *IP10a*, the revised version significantly improves the performance in many aspects: the predicted bulk modulus, elastic constants, and phonon frequencies are in excellent agreement with experiment, and the calculated defect energies are also more consistent with QM/MM predictions. Based on the new potential, we also calculated the phonon dispersion of CeO_2_ at 0 K (Figure 3). Experimental results are presented in markers for comparison, derived from reflectivity by inelastic neutron scattering by Clausen et al.^136^ and Marabelli and Wachter^137^ and Raman scattering by Nakajima et al.^82^ and Kourouklis et al.^138^ Our potential shows excellent agreement with the experimental measurements in terms of the phonon modes at the high symmetry points and acoustic mode dispersion, ensuring good accuracy for future study of thermodynamic properties of defective CeO_2_ using free-energy calculations.

A detailed comparison of QM/MM and M-L calculations of the vertical and adiabatic formation energies of holes and electrons in CeO_2_ is given in Table 4. Full computational details are given in Section S1.7 of the Supporting Information. For comparison, if we directly employ the Ce^4+^–O^2–^ short-range potential to model the localized electrons and holes without any correction (listed in the first column), as originally proposed by Freeman and Catlow,^100^ the bond lengths around the polarons and the band gap of CeO_2_ are significantly overestimated. Our corrected potential accurately reproduces the formation mechanisms of localized electrons and holes in CeO_2_ and gives a more reasonable band gap. This strategy could be a general approach and extended to other systems with localized charge carriers. The self-trapping energy of the localized electron is predicted to be −0.92 eV, compared with −0.61, −0.69, and −1.39 eV by the QM/MM approach using B97-2, PBE0, and BB1K functionals, respectively. Previous periodic DFT calculations reported −0.30 eV and −0.54 eV by HSE06 and PBE+U.^30^

**Table 4:** Vertical and Adiabatic Ionization Potentials (*I*_vertical_ and *I*_adiabatic_), Vertical and Adiabatic Electron Affinities (*A*_vertical_ and *A*_adiabatic_), Self-Trapping Energies (*E*_hole_^ST^ and *E*_electron_^ST^) of a Hole or an Electron, the Average First-Neighbor Bond Length around the Hole or Electron (*r*_Ce^4+^–O^1–^_ and *r*_Ce^3+^–O^2–^_), and the Band Gap (*E*_g_) of CeO_2_ Calculated by the M-L Approach and QM/MM Calculations^a^

	M-L (with modified shell charges)^b^	M-L (with modified shell charges and potentials)^b^	QM/MMB97-2^b^	QM/MM PBE0^b^	QM/MM BB1K^b^	Periodic HSE06^c^	Periodic PBE+U^c^
*I*_vertical_ (eV)	9.64	5.10	4.92	5.10	5.38		
*I*_adiabatic_ (eV)	7.11	3.78	4.35	4.41	3.80		
*E*_hole_^ST^ (eV)	–2.53	–1.32	–0.57	–0.69	–1.58		
*r*_Ce^4+^–O^1–^_ (Å)	2.533	2.451	2.447	2.451	2.452		
*A*_vertical_ (eV)	0.18	0.28	0.70	0.51	0.11		
*A*_adiabatic_ (eV)	1.44	1.20	1.31	1.20	1.50		
*E*_electron_^ST^ (eV)	–1.26	–0.92	–0.61	–0.69	–1.39	–0.30	–0.54
*r*_Ce^3+^–O^2–^_ (Å)	2.474	2.444	2.448	2.442	2.435	2.42	
*E*_g_ (eV)	8.08	4.82	4.22	4.59	5.27		

a In M-L calculations, the first column shows the results using modified shell charges for Ce^3+^ and O^1–^ but the same potential for Ce^4+^-O^2–^ interactions, while the second column shows the calculated results using *IP10b* presented in Table 3.

b Present work.

c Reference (30).

The predicted band gaps by the embedded-cluster approaches are higher than the experimental values for two reasons. First, despite the advantages in predicting bulk properties and formation energies of defects, most hybrid functionals significantly overestimate the band gap of CeO_2_.^36,37^ CeO_2_ is a strongly correlated *f* electron oxide, and the noncorrelated HF method predicts the band gap as 14.5 eV.^37^ As a result, the hybrid functional that includes a higher percentage of HF exchange usually provides a worse description of the band gap of CeO_2_. However, a certain percentage of HF exchange (usually 25%) is required to ensure the predictions of localized small polarons in CeO_2_, or both electrons and holes will become more delocalized even when trapped by defects, which is however contrary to experiment.^7,25,43^ Second, the overestimation of the band gap is partly due to the poor description of delocalized states and quantum confinement effects by the hybrid QM/MM embedded-cluster approach. Hence, it can be seen that the predicted “band gap” through *I*_vertical_ – *A*_vertical_ by the QM/MM method using the PBE0 functional (4.59 eV) is slightly higher than plane-wave periodic DFT calculations using VASP (4.38 eV) and the experimental measurement (4 eV). To counter these problems, we typically use the experimental band gap and calculated vertical ionization potential to determine the position of conduction band minimum (CBM) in QM/MM calculations.^56^

#### 3.2.1 Formation of Charged Defects in CeO_2_

**Figure 4:**
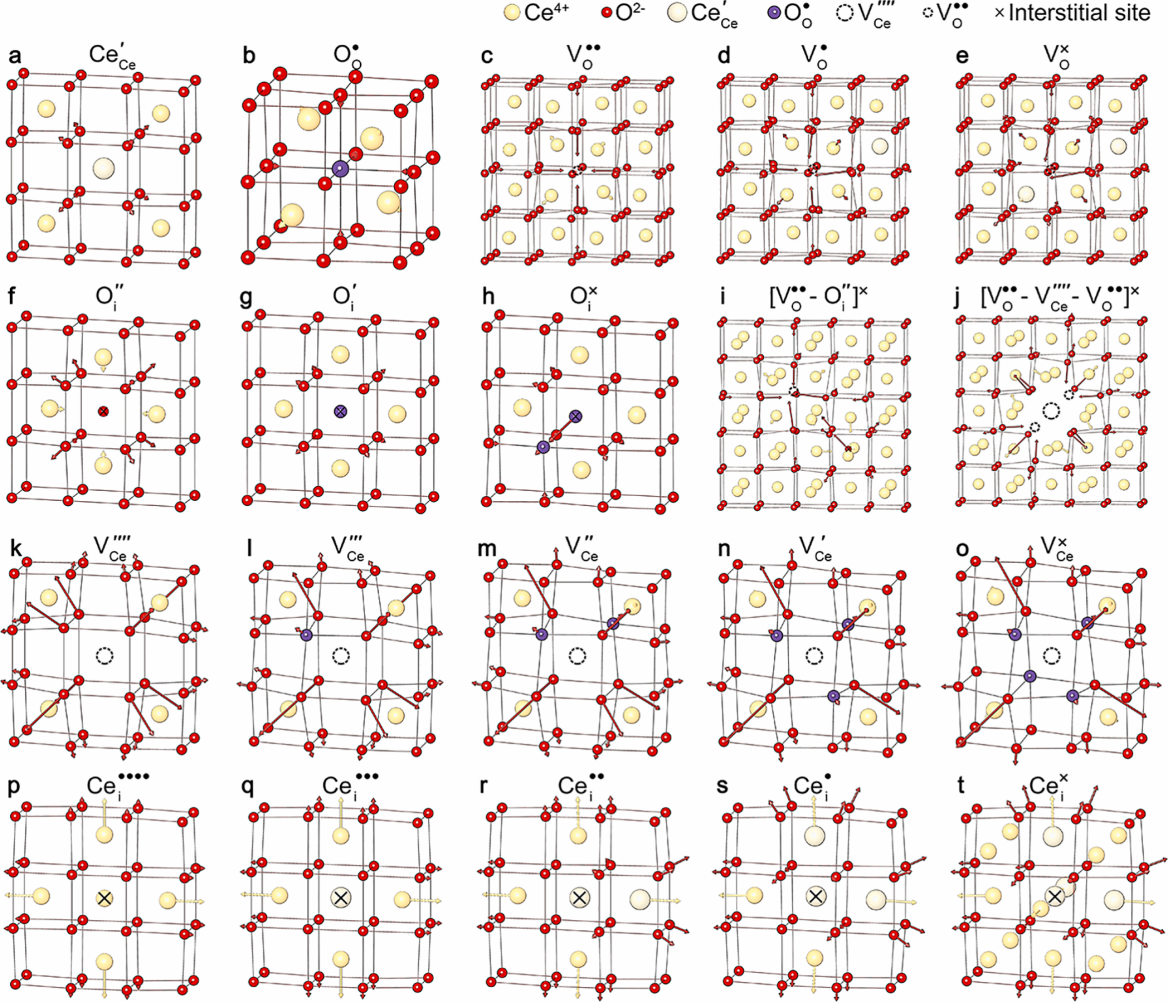
Defect-induced structural relaxation in CeO_2_ predicted by the Mott–Littleton approach. (a) Electron polaron Ce_Ce_^′^. (b) Hole polaron O_O_^•^. (c–e) Oxygen vacancies V_O_^*q*^, *q* = +2, +1, 0. (f–h) Oxygen interstitials O_i_^*q*^, *q* = −2, −1, 0. (i) Bound anion–Frenkel pair [V_O_^••^–O_i_^″^]^×^. (j) Bound Schottky trio [V_O_^••^–V_Ce_^⁗^–V_O_^••^]^×^. (k–o) Cerium vacancies V_Ce_^*q*^, *q* = −4, −3, −2, −1, 0. (p–t) Cerium interstitials Ce_i_^*q*^, *q* = +4, +3, +2, +1, 0. Arrows indicate directions and magnitudes of atomic displacements (magnified 6-fold to improve the clarity of images) upon relaxation.

Using the new IP, we modeled the formation of intrinsic point defects and defect pairs in CeO_2_ with the M-L and QM/MM approaches. The optimized structures of some typical defects in ceria obtained by the M-L approach are shown in Figure 4. The arrows indicate the directions and magnitudes (six times the actual length) of atomic displacements following the defect formation. We first consider the formation of atomic defects without trapped charge carriers. The formation of V_O_^••^ results in an inward displacement of the six nearest O^2–^ ions by 0.275 Å, compared with 0.223–0.245 Å in the QM/MM calculations. We note that the long-range polarization effect of V_O_^••^ is quite large, which causes the fourth, fifth, and sixth neighboring O^2–^ ions to move inward by 0.06 Å, 0.04 Å, and 0.02 Å, respectively. Unless a supercell with hundreds of atoms is used, periodic DFT calculations may have difficulties modeling such a long-range perturbation effect. Our embedded-cluster models explicitly considered this effect because sufficiently large active regions are used. The four neighboring Ce^4+^ ions displaced outward by 0.166 Å (corresponding to 7.1% of the equilibrium bond length), which agrees well with previous DFT calculations by Sun et al. (6.8% in HSE06 and 7.1% in PBE+U).^30^ For V_Ce_^⁗^, the largest displacement was on the eight coordinating O^2–^ ions, which move away from the defect center by 0.431 Å, while the surrounding Ce^4+^ ions are barely perturbed. The O_i_^″^ sits symmetrically in the center of the octahedral site. The O^2–^-O^2–^ bond length is calculated to be 2.50 Å (107.1% of the equilibrium interoxygen separation distance), which agrees well with the PBE+U prediction by Zacherle et al.^27^ (2.52 Å and 105.9% of the PBE+U bond length). Finally, the introduction of a Ce_i_^••••^ results in a strong repulsion to the nearest Ce^4+^ ions, forcing them to move away by 0.357 Å (0.32–0.324 Å by previous DFT+U predictions^25,28^ and 0.337–0.349 Å by our QM/MM calculations). Ce_i_^••••^ has much less influence on the O^2–^ ions (displaced only by 0.096 Å). Among these types of defects, O^2–^ ions are more easily affected by defect formation than Ce^4+^ ions (except Ce_i_^••••^, which is less common in CeO_2_ due to the relatively high formation energy). A detailed comparison of the structural relaxation due to defect formation is made in Table S3. Overall, M-L calculations show excellent agreement with previous periodic DFT and our QM/MM predictions of the defect structures.

**Figure 5:**
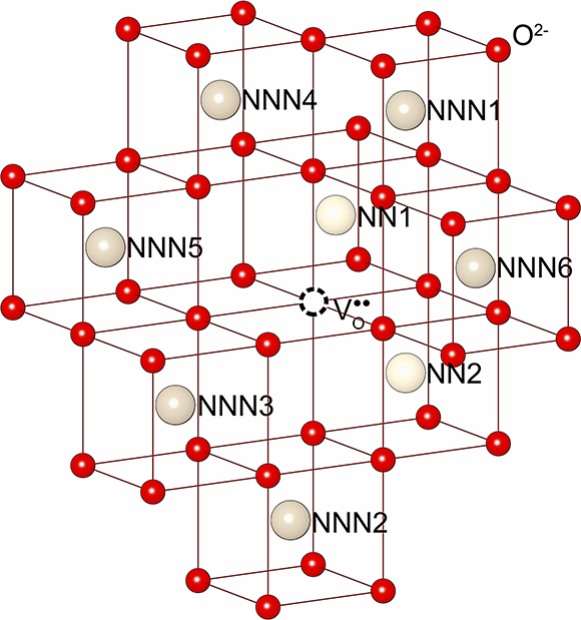
Electron polaron configurations in the charge-neutral oxygen vacancy [V_O_^••^–2Ce_Ce_^′^]^×^ in CeO_2_.

We also studied the formation energies of different charge states of native defects using the new IP and the embedded-cluster approaches. We started from the various possible configurations of polarons trapped by oxygen vacancies in bulk ceria. Previous DFT calculations have predicted that the oxygen vacancy becomes less stable when the two polarons locate beyond the second-neighbor sites.^29,42^ This conclusion is confirmed by our M-L calculations. The calculated formation energies of [V_O_^••^–Ce_Ce_^′^]^•^ in O-rich conditions when the Fermi level is at VBM are 0.049 eV, 0.046 eV, 0.306 eV, 0.267 eV, and 0.303 eV for the localized electrons at the first-, second-, third-, fourth-, and fifth-nearest neighbor sites, respectively. Hence, we only focus on the possible configurations within the second neighbor radius. For the charge-neutral oxygen vacancy [V_O_^••^–2Ce_Ce_^′^]^×^ formed in bulk ceria, the excess electrons will localize on two Ce sites (Ce_Ce_^′^). As shown in Figure 5 and Table 5, there are one NN-NN, three NN-NNN, and five NNN-NNN symmetrically unique configurations on which the two Ce_Ce_^′^ polarons can locate.^41^

**Table 5:** Calculated Formation Energies (eV) in O-Rich Conditions of Charge-Neutral Oxygen Vacancies Associated with Electron Polarons in Differing Configurations

Configuration	*d*_Ce^3+^–Ce^3+^_	M-L^a^	QM/MM PBE0^a^	QM/MM BB1K^a^	Periodic PBE+U^b^	Periodic PBE+U^c^	Periodic HSE06^c^	Periodic PBE+U^d^	Periodic PBE+U^e^	Periodic HSE06^e^	Periodic PBE0^f^
NN1-NN2	1N	3.657	4.04		2.91	2.50	3.73	2.22	2.70	4.09	3.84
NN1-NNN6	2N	3.681			2.95						
NN1-NNN1	1N	3.673			2.93						
NN1-NNN2	3N	3.564	3.70	2.92	2.85						
NNN1-NNN2	5N	3.596			2.92						
NNN1-NNN3	4N	3.604			2.85						
NNN1-NNN4	1N	3.578	3.77	3.02	2.85	2.43	3.63				
NNN1-NNN5	3N	3.579			2.86						
NNN1-NNN6	1N	3.604			2.92						

a Present work.

b Plane-wave PBE+U (U_Ce 4*f*_ = 4.5 eV) calculations using the 96-atom supercell.^29^

c Plane-wave PBE+U (U_Ce 4*f*_ = 5.0 eV) and HSE06 (25% HF) calculations using the 192-atom supercell.^31^

d Plane-wave PBE+U (U_Ce 4*f*_ = 5.0 eV and U_O 2*p*_ = 5.5 eV) calculations using the 96-atom supercell.^143^

e Plane-wave PBE+U (U_Ce 4*f*_ = 5.0 eV) and HSE06 (25% HF) calculations using the 96-atom supercell.^30^

f Plane-wave PBE0 (25% HF) calculations using the 96-atom supercell.^38^

The calculated formation energies of all nine configurations of [V_O_^••^–2Ce_Ce_^′^]^×^ in O-rich conditions are summarized in Table 5. In general, our simulations confirm that the excess electrons have lower energies when localized on the NNN site, consistent with other DFT predictions.^29,41,139^ The most stable configuration is the NN1-NNN2 with a formation energy of 3.564 eV, and the second stable NNN1-NNN4 configuration is only 0.014 eV higher in the formation energy. Our QM/MM calculations also favor the stabilization of the NN1-NNN2 configuration against the NNN1-NNN4 configuration, consistent with the M-L predictions. The tiny energetic deviations (<0.12 eV) among the formation energies of all the nine configurations suggest very close stabilities of different configurations, consistent with the previous periodic DFT+U study by Murgida et al..^29^ Because the adiabatic hopping barrier for Ce_Ce_^′^ in CeO_2_ is very low (0.15–0.2 eV^12,140,141^), the positions of polarons could change rapidly at elevated temperatures. Previous DFT calculations predict lower formation energy at the PBE+U level (2.2–2.95 eV)^26,29−31^ and slightly higher formation energy using HSE06 (3.63–4.09 eV^7,30,31^). Brugnoli et al.^38^ reported 3.84 eV for the formation energy of the NN1-NN2 configuration based on the periodic model using PBE0, compared with 4.04 eV from our QM/MM model using the same functional. Burow et al.^142^ conducted the periodic electrostatic embedded-cluster method (PEECM) using PBE0 but obtained a much lower formation energy (3.0 eV) than our QM/MM predictions and periodic calculations from Brugnoli et al.^38^ Our predictions at the PBE0 level of theory are also in line with the experimental heat of reduction of ceria, which is *ca*. 4.0 eV.^18,19^

**Figure 6:**
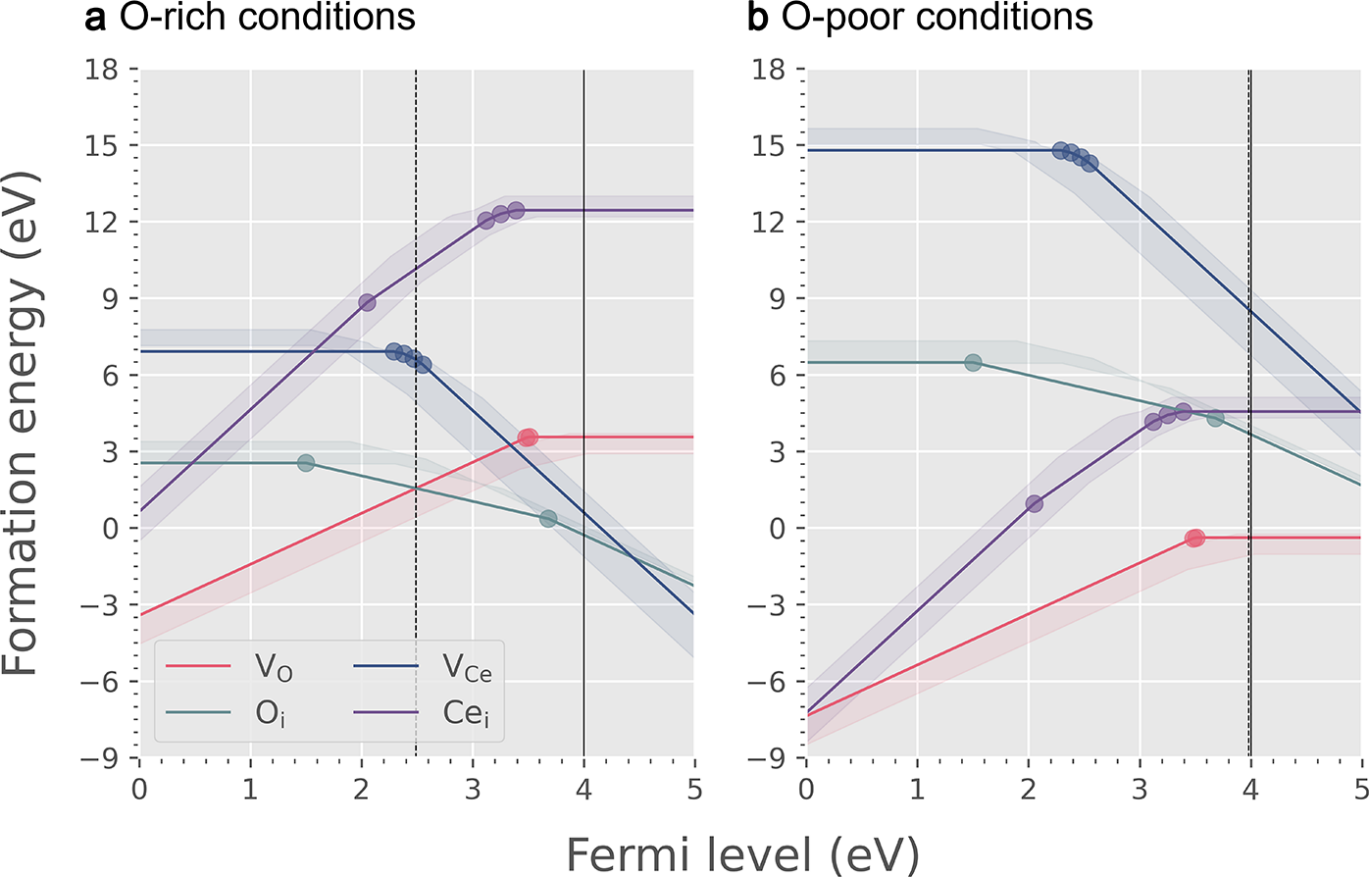
Calculated formation energies of intrinsic defects in CeO_2_ under different growth conditions using the M-L approach. Only the most stable charge states for each type of defect are shown. Solid circles indicate the thermodynamic transition levels. The lighter regions were determined by the minimum and maximum values obtained from B97-2, PBE0, and BB1K QM/MM calculations. The vertical solid lines show the position of the CBM according to the experimental band gap of 4 eV. The vertical dashed lines indicate the calculated self-consistent Fermi energy in each growth condition at 100 K, i.e., the low-temperature regime. (a) In O-rich/Ce-poor conditions. (b) In O-poor/Ce-rich conditions.

The same approach was used to investigate other charged defects with localized electrons and holes. Table 6 summarizes the calculated formation energies of all charge states of point defects studied by the M-L and QM/MM methods using our new IP. Figure 6 shows a direct comparison of the calculated formation energies under different conditions using the M-L approach and QM/MM calculations. These two embedded-cluster approaches present a high level of consistency in predicting the formation energies, confirming the success of our newly proposed fitting strategy for localized charge carriers. For the charge-neutral point defects compensated by electrons and holes, our predictions are in good agreement with periodic calculations. For highly charged defects, the long-range polarization effects should greatly contribute to the calculated formation energies as evaluated using the Jost correction in eq 9, which is −0.46 eV, −1.84 eV, −4.14 eV, and −7.37 eV for the ±1, ±2, ±3, and ±4 charge states, respectively. These effects were typically not explicitly considered in previous supercell models. Recently, there has been increasing awareness of the application of long-range electrostatic corrections toward more quantitative predictions of defect formation energies using supercell approaches.^50^ Apart from the long-range effect, it should also be noted that there are significant differences between the PBE+U and HSE06 predictions on V_O_^••^ and V_O_^*×*^ using the standard PAW method based on the same setting.^30^ Therefore, for these defects in higher charge states such as V_Ce_^⁗^ and Ce_i_^••••^, it is not surprising that PBE+U calculations might have several electron volts difference compared with hybrid functional predictions, although there is no corresponding reference data available.

**Table 6:** Calculated Formation Energies (eV) of Intrinsic Point Defects in CeO_2_ in O-Rich Conditions^a^

Defect reactions	M-L^b^	QM/MMB97-2^b^	QM/MM PBE0^b^	QM/MM BB1K^b^	Periodic HSE06^c^	Periodic PBE+U^c^	Periodic PBE+U^d^	Periodic PBE+U^e^
(a) Oxygen vacancy								
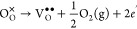	4.57	4.59	4.61	3.45	5.0	4.3	4.7	4.43
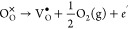	4.05	3.52	3.84	2.89	4.5	3.4	4.13	3.66
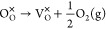	3.56	3.11	3.70	2.92	4.09	2.7	3.16	2.8
(b) Oxygen interstitial								
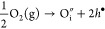	7.72	7.86	7.87	8.08			7.40	4.41
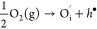	4.04	5.28	5.14	4.81			4.49	3.60
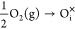	2.54	3.39	3.17	2.51			1.82	2.79
(c) Cerium vacancy								
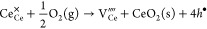	16.60	14.87	16.01	17.44			9.0	7.39
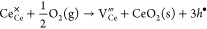	14.05	12.46	13.43	14.35			8.0	6.32
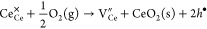	11.58	11.00	11.81	12.24			7.1	6.52
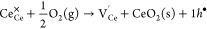	9.20	8.71	9.31	9.20			5.8	6.81
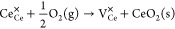	6.91	7.35	7.77	7.14			5.0	7.39
(d) Cerium interstitial								
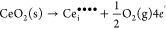	16.63	17.60	16.94	15.48			13.45	14.46
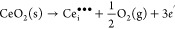	14.68	15.86	15.41	14.02			12.95	13.55
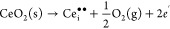	13.8	14.62	14.43	13.17			12.75	12.57
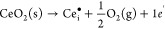	13.05	13.44	13.71	12.61			12.05	11.69
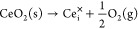	12.44	12.64	13.00	12.19			11.69	10.9

a Reaction energies are reported assuming holes formed at the VBM and electrons at the CBM (using the calculated VBM at each level of theory and experimental 4 eV band gap for obtaining CBM).

b Present work.

c Plane-wave PBE+U (U_Ce 4*f*_ = 5.0 eV) and HSE06 (25% HF) calculations using the 96-atom supercell.^30^

d Plane-wave PBE+U (U_Ce 4*f*_ = 4.0 eV and U_O 2*p*_ = 4.0 eV) calculations using the 96-atom supercell with a nonlinear core-corrected (NLCC) norm-conserving pseudopotential for Ce.^28^

e Plane-wave PBE+U (U_Ce 4*f*_ = 5.0 eV) calculations using the 96-atom supercell with PAW.^27^

Our M-L predictions of the formation energies of charge-neutral defect pairs and trios are also in good agreement with QM/MM predictions. When an anion–Frenkel pair is formed, a lattice oxygen ion occupies an octahedral interstitial site, leaving an oxygen vacancy. The formation energy is predicted to be 2.15 eV per defect (4.30 eV for the total defect reaction) by our potential model at infinite separation (2.23 eV, 2.24 eV, and 1.77 eV at the QM/MM B97-2, PBE0, and BB1K levels of theory, respectively, 2.07–2.08 eV by periodic PBE+U calculations,^27,28^ and 1.83 eV by experiment^144^). In our M-L calculations, we observed the recombination behavior of bound anion–Frenkel pairs in the nearest (2.34 Å) and next-nearest (4.47 Å) separations. The first stable anion–Frenkel pair has a separation distance of 5.88 Å with a total formation energy of 3.85 eV (viewed as a defect cluster), yielding a binding energy of 0.45 eV. The second stable pair has a slightly higher formation energy of 3.98 eV due to the larger separation (7.01 Å). Our M-L calculations predict a similar critical stabilization distance of the bound anion–Frenkel pair as reported by Huang et al.^28^ using periodic PBE+U calculations (5.95 Å). The M-L calculated formation energy of the unbound Schottky trio [V_Ce_^⁗^] + 2[V_O_^••^]) is of 3.25 eV per defect, compared with 2.69 eV by B97-2, 3.08 eV by PBE0, and 2.78 eV by BB1K functionals in QM/MM. Previous periodic PBE+U studies reported 2.29 eV by Zacherle et al.^27^ According to our M-L calculations, Schottky trios can form stable bound complexes with formation energies of 5.18 eV, 5.22 eV, and 5.86 eV for two oxygen vacancies aligned along the ⟨111⟩, ⟨110⟩, and ⟨100⟩ directions, respectively, with respect to the cation vacancy. The corresponding binding energies are quite high due to the high charges and close proximity of the cation and anion vacancies, whose distances are 4.58 Å, 4.53 Å, and 3.89 Å, respectively. Our predicted formation energy for the bound Schottky trio is slightly higher than other PBE+U calculations of 3.66 eV by Keating et al.^25^ and 3.86 eV by Huang et al.^28^ The calculated formation energy for the interstitial disorder ([Ce_i_^••••^] + 2[O_i_^″^]) is 5.36 eV by the M-L approach, also in good agreement with the predictions of 5.77 eV, 5.56 eV, and 5.21 eV at the QM/MM B97-2, PBE0, and BB1K levels of theory. We also predicted a higher formation energy for the cation–Frenkel pair ([V_Ce_^⁗^] + [Ce_i_^••••^]) than other periodic PBE+U calculations (8.62 eV compared with 5.8–6.3 eV^26−28^). However, the M-L prediction agrees well with our QM/MM results (8.24 eV, 8.48 eV, and 8.46 eV at the B97-2, PBE0, and BB1K levels of theory, respectively).

The anion–Frenkel pair ([V_O_^••^] + [O_i_^″^]) is predicted to be the dominant defect pair in bulk ceria, consistent with a recent neutron scattering study.^16^ This behavior is similar to other cubic fluorite crystals such as UO_2_ and cubic ZrO_2_.^72,145^ Oxygen vacancy formation is of crucial importance in bulk ceria, which can repeatedly absorb and release oxygen in catalytic reactions through the Mars–van Krevelen mechanism.^9,10^ Furthermore, the V_O_^••^ migration barrier is very low (0.53 eV predicted by our potential, 0.5–0.6 eV by previous DFT and experimental studies^30,85,146−148^), which could be further reduced by the surrounding Ce_Ce_^′^ polarons on the surfaces,^12,140^ ensuring good ionic conductivity in SOFC applications. All these features make this material versatile in energy and catalytic applications.

The energetic difference in predictions between the embedded-cluster approaches and periodic PBE+U results could mainly originate from the differences in DFT functional and long-range polarization effects. As illustrated before, the periodic PBE+U approach may not be accurate enough for the formation energy of highly charged defects, while our QM/MM calculations that employ hybrid functionals and consider long-range polarization should be more reliable and target the defect formation at the dilute limit. Overall, *IP10b* accurately describes defect structures and formation mechanisms in ceria, which could be useful for investigating more complex defect clusters and partially reduced phases that are computationally too expensive for first-principles calculations. Moreover, our potential model for modeling holes and electron polarons is highly transferable. Using the same correction approach, one can reproduce the predictions of other hybrid functionals.

#### 3.2.2 Thermodynamic Defect Processes in Bulk CeO_2_

**Figure 7:**
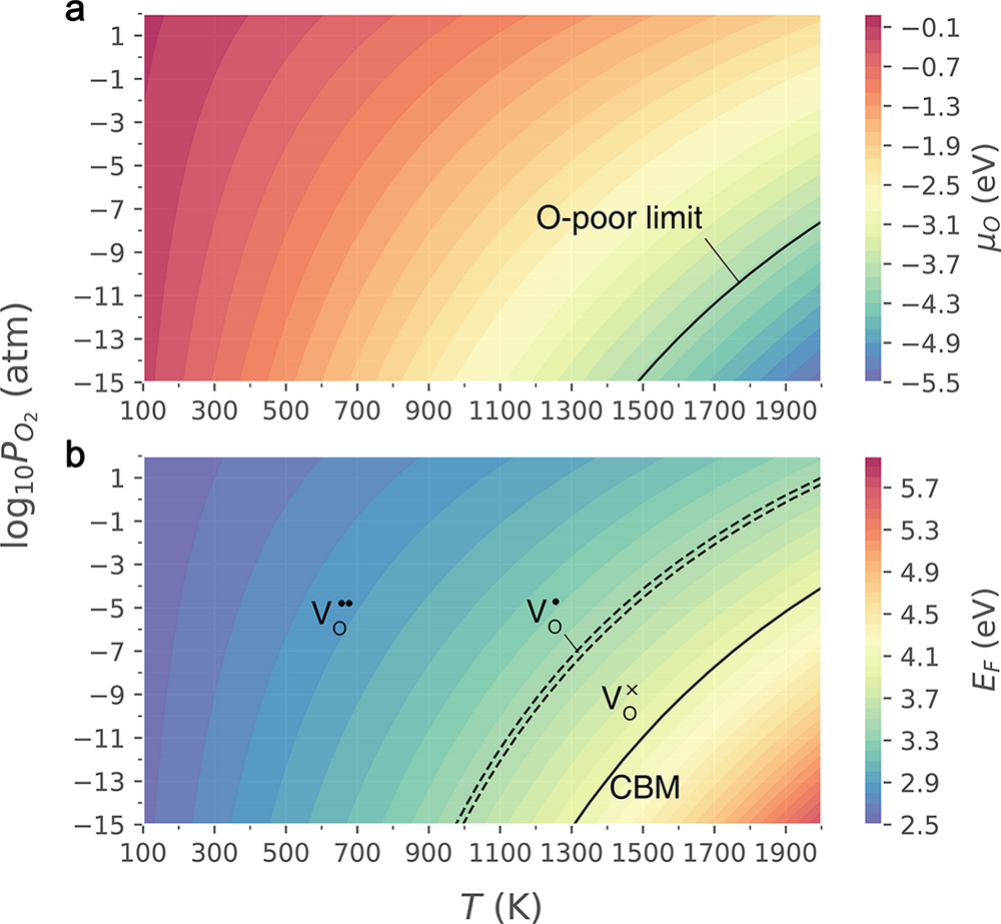
Variations of the (a) oxygen chemical potential and (b) calculated self-consistent Fermi level relative to VBM with temperature and oxygen partial pressure.

The formation energy of a point defect depends on the position of the Fermi level (electronic chemical potential) and chemical potentials of exchanged species according to eq 8, which will be further affected by conditions, especially oxygen partial pressure *P* and temperature.^107^ As summarized by Reuter and Scheffler,^149^ the relationship between the oxygen chemical potential μ_O_(*T*, *P*) and oxygen partial pressure *P* can be obtained by
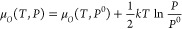
29
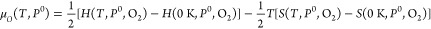
30where *P*^0^ = 1 atm is typically defined as the zero state,  eV, *H* is the enthalpy, and *S* is entropy. Using the experimental enthalpy and entropy data,^150^ we calculated the value of μ_O_ at various reaction conditions. As shown in Figure 7a, μ_O_ decreases with increase in temperature or decrease in *P*. In the case of CeO_2_, an extreme reducing condition (e.g., *P* = 2.3 × 10^–10^ atm at 1800 K) is required to reach the O-poor limit of μ_O min_ = −3.94 eV at which Ce_2_O_3_ becomes more stable than CeO_2_. In realistic systems, the formation energies of defects always lie between the two limits shown in Figure 6.

The Fermi level of CeO_2_ is in turn also affected by temperature and oxygen partial pressure.^107^ Variation of the Fermi level influences further the dominant defect type, energetics of charge states, and concentrations of defects in solids. The self-consistent Fermi energy (shown in Figure 7b) was determined using the SC-FERMI code^151^ based on the M-L defect formation energies and density of states of the pristine CeO_2_ (obtained using VASP with PBE0 functional, where the unoccupied states were shifted downward to reproduce the experimental band gap of 4 eV). In the O-rich limit, the Fermi level lies *ca*. 2.5 eV above the VBM and favors the formation of doubly ionized V_O_^••^. These vacancies could be compensated by acceptor defects such as O_i_^″^ and O_i_^′^ or electrons that are not trapped at the vacancy sites. With the increase in temperature or decrease in oxygen partial pressure, the Fermi level rises toward the CBM, which could further support the stabilization of V_O_^•^ and V_O_^×^ with trapped electron polarons. An electrical conductivity study by Tuller and Nowick^44^ on single-crystal CeO_2–*x*_ samples also concluded that V_O_^••^ is dominant at small *x* in CeO_2–*x*_ (*x* < 10^–3^), with a transition to singly ionised V_O_^•^ at greater *x* under reduced *P*, confirming our predictions. The thermodynamic transition level *ε*(*q*_1_/*q*_2_) is defined as the energy of an adiabatic ionization process changing the charge state *q*_1_ of the defect to another charge state *q*_2_. It can be obtained as the electron Fermi level where the formation energies of two charge states of a point defect are equal or using the following formula:^39^
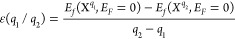
31where *E*_*f*_(X^*q*^, *E*_*F*_ = 0) is the formation energy of the defect X with the charge state *q* when the Fermi level is at the valence band maximum (VBM, *E*_*F*_ = 0). The thermodynamic transition level indicates the relative stability of the two charge states. When *E*_*F*_ < *ε*(*q*_1_/*q*_2_), the charge state *q*_1_ is stable and *vice versa*. The calculated thermodynamic transition levels of oxygen vacancy are shown as dashed lines in Figure 7b. In our M-L calculations, *ε*(+2/+1) *=* 3.48 eV and *ε*(+1/0) = 3.51 eV above the VBM. Sun et al.^30^ have compared DFT PBE+U and HSE06 predictions using the supercell approach with an appropriate correction scheme on charged defects. They also obtained high transition levels for oxygen vacancies using the HSE06 functional (*ε*(+2/+1) *=* 3.1 eV and *ε*(+1/0) = 3.2 eV), but their PBE+U calculations yielded much lower values of *ε*(+2/+1) *=* 1.45 eV and *ε*(+1/0) = 1.65 eV. This difference could originate from the accuracy of the predicted band gap from the DFT functionals.

Our previous discussion focused on the equilibrium-state defect formation in bulk CeO_2_ at the dilute limit, without considering the extrinsic doping and surface effects. Even trace dopants, or impurities can considerably affect the position of the Fermi level and defect formation.^26,70,152^ Further, we should note that the formation energies of V_O_^×^ on or near the surfaces are much lower than their counterparts in bulk, which could be rationalized by a decrease in the coordination of atoms exposed at the topmost surface layer. Previous PBE+U calculations reported an energetically favorable formation of V_O_^×^ in the subsurface layer of CeO_2_(111) with a formation energy of *ca*. 1.8 eV compared with 2.85 eV in bulk.^24,153^ The formation energies of V_O_^×^ on less stable CeO_2_(110) and CeO_2_(100) surfaces are predicted to be even lower by Ganduglia-Pirovano et al.^24^ and are 1.06 and 1.35 eV, respectively. Such low formation energies of V_O_^×^ should result in nonnegligible shifts of transition levels toward the VBM and would favor the trapping of excess electrons near the surfaces, as experimentally captured by STM images.^40,43^ This observation is, in fact, quite general. For example, recently, Wang and Yin^154^ also found that iodine vacancies in CH_3_NH_3_PbI_3_ formed in the bulk and on the surface have significant differences in the transition level and effects on photovoltaic performance. Returning to CeO_2_, reduction in practice always starts from the near-surface layers, resulting in the localization and segregation of excess electrons, which could lead to further reconstruction into alternative ordered phases such as CeO_1.68_, Ce_7_O_12_, Ce_11_O_20_, and Ce_3_O_5_.^16,110,155,156^ Trace dopants or unintended impurities including divalent^157^ and trivalent^158^ metal ions and surface absorbates such as fluorine^159^ and hydroxyls^160^ could also become the charge-compensating species for V_O_^••^ and have a large impact on the Fermi level and defect concentrations. Our calculations show that, with the increase in temperature or decrease in oxygen partial pressure, the Fermi level rises in bulk CeO_2_, and singly ionized V_O_^•^ and charge-neutral V_O_^×^ become dominant before the formation of ordered reduced phases. A complete understanding of the partial reduction behavior of ceria requires further investigation using, e.g., free energy calculations, Monte Carlo, or molecular dynamics techniques^161−164^ to consider explicitly entropic effects of defect formation and defect–defect interactions, which will be investigated in future research based on our new potential.

## 4 Conclusions

We have proposed a new strategy for deriving shell-model interatomic potentials based on hybrid QM/MM embedded-cluster calculations of ionic polarizabilities, defect structures, and formation energies. A new potential has been developed for CeO_2_, with a distinctive performance in predicting the structure, elastic, dielectric, defect, surface, phonon, and thermodynamic properties compared with experimental measurements and *ab initio* calculations. In particular, the calculated structures and formation energies of polarons, various charged states of native defects, and defect pairs in CeO_2_ achieved a high level of consistency between the M-L calculations and QM/MM results. Benefiting from our new potential, one could provide more insights into the complex defect chemistry of CeO_2_. These developments will enable future work on defect formation at elevated temperatures using free energy calculations; partial reduction and nonstoichiometry based on molecular dynamics and Monte Carlo simulations; and the role of surface defects in catalytic reactions using QM/MM techniques. This novel strategy for developing robust shell-model potentials capable of predicting accurate defect properties could be further extended to other ionic systems.

## Supporting Information Available

Additional information on the methodological details of plane-wave DFT calculations, interatomic-potential-based calculations, QM/MM interface treatment, Mott–Littleton and QM/MM calculations on several properties of ceria; supplementary data on variants of Buckingham potentials with different ρ values and their performances, shell-model potential parameters in the literature, comparison of atomic displacements due to defect formation, and the new potential *IP10b* in GULP-readable format (PDF)
