# Supplementary material for: Toward a Consistent Prediction of Defect Chemistry in CeO2
Source: Chem Mater. 2022 Dec 21;35(1):207–27. doi: 10.1021/acs.chemmater.2c03019 (PMC9835833; doi:10.1021/acs.chemmater.2c03019)
Supplement: Supplementary file 1 — cm2c03019_si_001.pdf [file cm2c03019_si_001.pdf]

Supporting Information for

Towards A Consistent Prediction of Defect Chemistry in CeO_2

Xingfan Zhang[1], Lei Zhu[1], Qing Hou[1, 5], Jingcheng Guan[1], You Lu[2], Thomas W. Keal[2], John Buckeridge[3], C. Richard A. Catlow[1, 4, *], and Alexey A. Sokol[1, *]

1 Kathleen Lonsdale Materials Chemistry, Department of Chemistry, University College London, London WC1H 0AJ, United Kingdom.

2 Scientific Computing Department, STFC Daresbury Laboratory, Warrington, Cheshire WA4 4AD, United Kingdom.

3 School of Engineering, London South Bank University, London SE1 OAA, United Kingdom.

4 School of Chemistry, Cardiff University, Park Place, Cardiff CF10 1AT, United Kingdom.

5 Institute of Photonic Chips, University of Shanghai for Science and Technology, Shanghai 200093, China

* c.r.a.catlow@ucl.ac.uk; a.sokol@ucl.ac.uk

Table of contents

1 Supplementary methodological details 2

1.1 Plane-wave DFT calculations 2

1.2 Interatomic-potential-based calculations of oxygen vacancy migration 3

1.3 Interatomic-potential-based surface calculations 3

1.4 QM/MM interface treatment 3

1.5 Calculations of in-lattice and gas-phase ionic polarisabilities 4

1.6 Effects of parameter ρ in the Ce^{4+}-O^{2-} Buckingham potential 5

1.7 Calculations of ionisation potentials, electron affinities, and band gap using Mott-Littleton and QM/MM approaches 6

2 Supplementary data 7

3 Interatomic potential parameters for CeO_2 (*IP10b*) in GULP-readable format..10

4 References...12

1 Supplementary methodological details

1.1 Plane-wave DFT calculations

A plane-wave basis set with a cut-off energy of 800 eV and projector augmented wave method (PAW) was used, in which the Ce (5*s*, 5*p*, 4*f*, 5*d*, 6*s*) and O (2*s*, 2*p*) states were treated explicitly as valence states.[1] The bulk and surface calculations were performed at several levels of theory for comparison. The PBE exchange-correlation functional[2] and its solids-corrected version (PBEsol)[3] were used with a Hubbard correction scheme (DFT+U, $U_{Ce\ 4f}$ = 5 eV) for the Ce 4*f* orbitals, which has proved to describe accurately the localised nature of the *f* electrons in CeO_2.[4-6] Hybrid functionals were also employed, including PBE0, PBEsol0, and HSE06 with 25% non-local Hartree-Fock (HF) exchange.[7-8] The convergence tolerance of electronic structure calculations was set to 10^{-7} eV, and structural optimisations were regarded as converged when the force on each ion had decreased to less than 10^{-2} eV Å^{-1}. Bulk calculations were performed based on the primitive cell of CeO_2 using an 11 × 11 × 11 Monkhorst-Pack *k*-point mesh.

The three low-index surfaces of CeO_2 ─ (100), (110), and (111) ─ are Tasker Type III (polar), Tasker Type I (nonpolar), and Tasker Type II (quadrupolar) surfaces, respectively.[9-10] The pristine (100) surface is known to be intrinsically unstable due to the presence of a dipole moment perpendicular to the surface. Polar-induced surface reconstruction occurs naturally to neutralise the dipole moment. For the (100) model, we followed the conventional approach using an O-terminated model with half of the surface oxygen atoms moved to the opposite surface of a slab cut from bulk.[11] To calculate the surface relaxation and surface energies, we employed periodic slab models with 15 atomic layers for (110), 15 double layers for (100), and 15 triple layers for (111), which are thicker than the minimum numbers of layers for obtaining converged surface energies (< 0.01 J m^{-2}) and improve the convergence of the surface energies, ionisation potentials and ionic displacements approximately by one order of magnitude. We fixed the central (double/triple) layers in each model and allowed other atoms to relax during the structural optimisation. A 30-Å-thickness vacuum layer was used in each model to minimise artificial interactions between slabs periodically repeated in the direction normal to the surface. The thickness of the slab and vacuum layers was tested to ensure converged surface energies. The electronic structures of the (100)-p(2 × 2), (110)-p(2 × 1), and (111)-p(1 × 1) surface models were obtained using 7 × 7 × 1, 5 × 7 × 1, and 7 × 7 × 1 *k*-point sampling grids across the 1st Brillouin zone, respectively. The surface energy is calculated as:

$$E_{surf} = \frac{E_{slab} - E_{bulk}}{2S} \tag{1}$$

where E_{slab} and E_{bulk} are the total energies of the optimised slab model and the bulk CeO_2 containing the same number of atoms, respectively, and S is the calculated surface area.

1.2 Interatomic-potential-based calculations of oxygen vacancy migration

The migration barrier of oxygen vacancy $E_{V_O}^{\mathrm{migration}}$ along the CeO_2<100> pathway was found using the rational function optimisation (RFO) method[12], as implemented in GULP[13]. Previous DFT studies have shown that the saddle point occurs halfway between two adjacent oxygen sites in the migration of the doubly ionised oxygen vacancy.[14-15] Hence, in the initial model, we created two adjacent oxygen vacancies with one migrating oxygen ion at the halfway point. Geometry optimisation using the RFO method is then performed to obtain the transition state structure and energy. Finally, the migration barrier is calculated by the energy difference between the saddle point and the optimised oxygen vacancy formation energy."

1.3 Interatomic-potential-based surface calculations

IP-based surface calculations were performed based on the 2D-periodic model with the two-region approach implemented in GULP. Our (100), (110), and (111) surface models have a thickness of *ca.* 62 Å, 50 Å, and 55 Å in the *c* direction, respectively, ensuring good convergence of the calculated surface energies to 0.01 J m^{-2}. The top half of layers in each model were allowed to relax during optimisation, whereas both cores and shells in the other bottom half of layers were fixed to reproduce the bulk environment. The three-region model that fixes the middle layer of the slab and allows both sides of surfaces to relax was also employed to check consistency between the two approaches, which reproduced the predicted structures and surface energies from the two-region models.

1.4 QM/MM interface treatment

In a well-constructed QM/MM model, ions in the QM region should behave in the same way as in the full QM model. In one aspect, the atoms on an optimised lattice should have zero forces (energy gradients). If the MM model is well parameterised, the lattice positions of both fully QM and fully MM

models would be close, meaning that any residual forces on a well-constructed QM/MM model should be minimal. The second conjecture is that symmetry-equivalent ions should have identical electrostatic potentials at their nuclei. Such potentials however are not always available from molecular QM calculations (depending on the available code). We therefore introduce this constraint by a requirement for deep core levels of such symmetry equivalent ions to equalise.

Conventionally, the interface is described by a large-core ECP on the cation sites. However, this approach could generate large gradients on ions and result in errors in energy calculations.[16] We therefore introduce a constraint by a requirement for deep core levels of such symmetry-equivalent ions to equalise. Implementing these ideas for QM/MM model is thus very much in the spirit of the mean-field theory. Instead, we used a specially designed local pseudopotential on the cationic sites in the form of a linear combination of three Gaussian functions.[16] As we do not attempt to model accurately chemical interactions across the QM/MM interface, reproducing exchange and correlation potentials, we avoid model over-parameterisation by sticking with the simplest model local pseudopotential, as first proposed in Ref. [16]. The main intent in choosing this shape is to account for the Coulomb screening of cation nuclei within the cation core while providing enough variational freedom for the description of the short-range repulsive potential.

The parameters of the three Gaussian functions were carefully determined by combining a global search on large parametric space and a refinement procedure using Powell's method[17] as implemented in our open-source code FIT_MY_ECP (https://www.github.com/logsdail/fit_my_ecp) combined with Tcl-ChemShell[18]. In the fitting of the interface pseudopotential, we mainly considered four observables, targeting the sources of errors as mentioned above, including the average gradients ($Gnorm$) in the QM, interface, and MM regions (which compose of the active region in the structural minimisation), and the spread of the O_{1s} core level ($Spread$). The target function F in the fitting is defined as the weighted sum of squares of the four descriptors:

$$F = wt_1\, Gnorm_1{}^2 + wt_2\, Gnorm_2{}^2 + wt_2\, Gnorm_3{}^2 + wt_4\, Spread^2 \quad (2)$$

We combined global (linear search) and local minimisation (Powell's method) approaches to minimise the target function, therefore reducing the gradients in the three regions and aligning the O_{1s} core levels to improve the QM/MM embedded-cluster model. The fitting was conducted recursively for the three Gaussian functions until no lower F could be obtained.

1.5 Calculations of in-lattice and gas-phase ionic polarisabilities

For calculating the intrinsic in-lattice ionic polarisability, only one QM atom (Ce^{4+} or O^{2-}) is required in the embedded-cluster environment. Electronic structure calculations were performed in the QM region using hybrid DFT functionals and higher-level coupled-cluster methods using NWChem.[19] The Def2-TZVP[20-21] basis set was used for both Ce and O atoms. Two point charges were put on opposite sides of the embedded-cluster model to form a uniform electric field across the central QM atom, with a separation distance of 200 Å (note that only one Cartesian direction needs to be probed for a cubic system like fluorite lattice). Ionic polarisabilities can then be obtained either as the first derivatives of the induced dipoles on the embedded QM atom or the second derivatives of the energy with the field strength. Shell relaxation is forbidden in these calculations so that the choice of IPs in the MM region will not affect the polarisability results; the MM ions produce only a Madelung field on the QM atom while the interface ions confine the QM electronic density.

The gas-phase ionic polarisabilities were calculated using the same approach but without the embedded-cluster environment (i.e., the system is composed of a QM atom in between two point charges).

1.6 Effects of parameter *ρ* in the Ce^{4+}-O^{2-} Buckingham potential

Figure S1 shows how parameter ρ affects the predicted properties of CeO_2. The dark grey horizontal lines represent the reference data from experiment or DFT calculations at the PBE0 level of theory. The lighter grey areas define the reference regions for defect energies, considering QM/MM predictions using three hybrid exchange and correlation functionals, B97-2, PBE0, and BB1K. In general, with an increase in ρ, the predicted bulk modulus, elastic constants, phonon frequencies, Born effective charges of Ce^{4+}, formation energies of Schottky trio and anion-Frenkel pair, $V_O^{\bullet\bullet}$ migration barrier, and (100) and (111) surface energies gradually decrease, whereas the lattice energy, O^{2-} Born effective charges, the formation energy of cation-Frenkel pair, and (110) surface energies increase. If we only consider the bulk properties that are normally used as fitting observables, a larger value of ρ such as ρ = 0.37 Å could be the result obtained by the least-squares procedure. However, if we further consider the defect and surface properties, those IPs with a larger value of ρ produce inaccurate results for ceria. In particular, we found that using ρ > 0.37 Å could result in imaginary frequencies at some *k*-points and problems in the calculations of $V_O^{\bullet\bullet}$ migration. Here, we highlighted some crucial factors for determining the best ρ:

(1) The CeO_2 (100) polar surface energy should be much higher than that of the nonpolar CeO_2 (110) surface as indicated by DFT calculations.[11, 22] The IPs using ρ > 0.365 Å predict the wrong trend.

(2) Due to the oxygen reservoir nature of ceria, the anion-Frenkel pair should be the dominant defect rather than the Schottky trio. This conclusion is confirmed by previous experimental and theoretical research and is consistent with our QM/MM calculations.[23-24] Other cubic fluorite crystals such as ZrO_2 and UO_2 also show the same feature.[25-26] Only IPs with $\rho \leq 0.355$ Å can correctly reproduce this characteristic.

(3) IPs with 0.35 Å ≤ ρ ≤ 0.36 Å reproduce more accurate phonon frequencies.

(4) The $V_O^{\bullet\bullet}$ migration barrier along the <100> direction is consistently reported to be 0.5-0.6 eV by experimental and theoretical studies,[14-15, 27-29] with a prior choice of ρ = 0.345 Å and 0.349 Å.

(5) QM/MM calculations of accurate defect energies of point defects will also support the preference for lower values of ρ, but will, however, not be used for the fitting, and rather reserved for validation of our MM results later.

By systematically considering these properties, *IP10a* with ρ = 0.349 Å is the best Buckingham potential for CeO_2, which was further used in QM/MM defect calculations.

1.7 Calculations of ionisation potentials, electron affinities, and band gap using Mott-Littleton and QM/MM approaches

In M-L and QM/MM calculations, we calculate the fundamental band gap, E_g, as the energy difference between the vertical ionisation potential (I_{vertical}) and vertical electron affinity (A_{vertical}), *i.e.*, $E_g = I_{\mathrm{vertical}} - A_{\mathrm{vertical}}$, which is consistent with experimental measurement.[30] I_{vertical} and A_{vertical} can be obtained by introducing a positive or negative charge into the system and calculating the energy difference between the N-electron ground state and N ± 1-electron state, respectively.

Whereas in QM/MM calculations, the change in the charge state of the QM cluster is straightforward, in M-L calculations, the shell charge of the central ions needs to be modified in the embedded-cluster model, as described by Freeman and Catlow[31]. For example, the shell charge of O^{2-} is changed from -3.297314 e to -2.297314 e (core charge keeps 1.297314 e) and the ion becomes O^{1-}. Modelling vertical processes, we only allow the relaxation of shells (to represent the electronic redistribution when a charge is added to or removed from the system), while the positions of atoms represented by cores are fixed. By contrast, to obtain the adiabatic ionisation potential

($I_{\text{adiabatic}}$) and electron affinity ($A_{\text{adiabatic}}$), both shells and cores are allowed to relax fully upon optimisation. Furthermore, M-L calculations do not account for the intraatomic energy difference between the N-electron ground state and N ± 1-electron state. The latter term can be approximated by the fourth ionisation potential of Ce atom (I_{Ce}^{4} = 36.762 eV[32]) and second electron affinity of O atom (A_{O}^{2} = 8.14 eV).[31]

In our improved method, we further consider the change in short-range interactions when forming O^{1-} and Ce^{3+}, by fitting the potential parameters to reproduce the QM/MM calculated structures and formation energies of electron and hole polarons. Our revised approach significantly improves the description of the band gap in the M-L method (from 8.08 eV to 4.82 eV), as well as gives a much more accurate description of localised charge carriers in the system, as shown in Table 4 of the main text.

2 Supplementary data

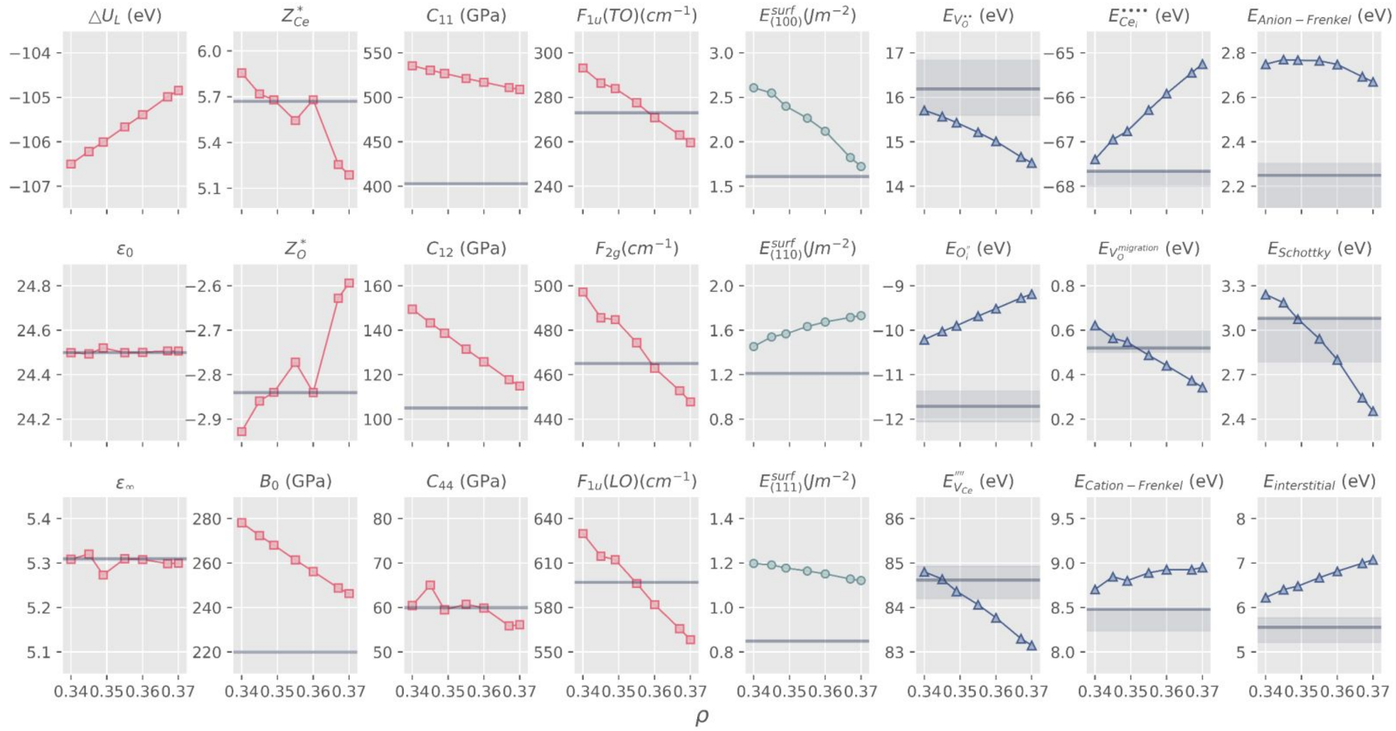

Figure S1. Performance of candidate IPs in describing the properties of CeO_2 as a function of parameter ρ in the Ce^{4+}-O^{2-} Buckingham potential. The dark grey horizontal lines represent the selected reference data from experiment or DFT calculations at the PBE0 level of theory. The lighter grey regions in the graphs of defect energies define the minimum and maximum values obtained from QM/MM calculations using the B97-2, PBE0, and BB1K functionals.

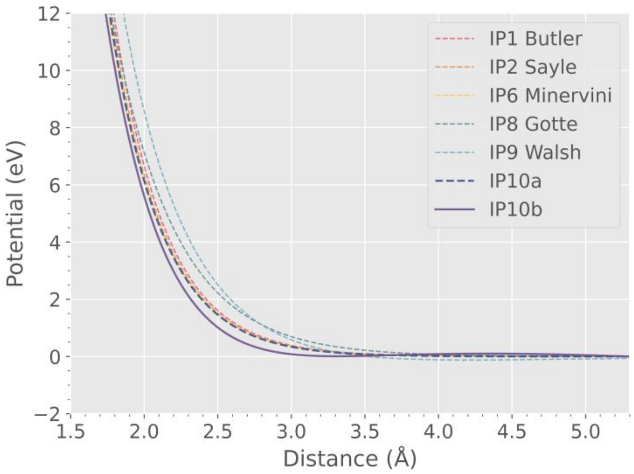

Figure S2. The Ce^{4+}-O^{2-} short-range potentials developed in this work compared with previous interatomic potentials in the literature.

Table S1. Previously developed shell-model IPs for CeO_2 from the literature. Parameters provided here are defined in eq. (2-3) in the main text.

IP	Interaction	A (eV)	ρ (Å)	C_6 (eV Å^6)	Ion	Y (e)	k_2 (eV Å^{-2})	k_4 (eV Å^{-4})
IP1 Butler1983[33]	O^{2-}-O^{2-}	22764.3	0.149	43.83	O^{2-}	-2.83	257.89	
	Ce^{4+}-O^{2-}	1986.83	0.35107	0	Ce^{4+}	9.38	201.62	
IP2 Sayle1994[34]	O^{2-}-O^{2-}	22764.3	0.149	43.83	O^{2-}	-6.1	419.874	10000
	Ce^{4+}-O^{2-}	1986.83	0.35107	20.4	Ce^{4+}	7.7	291.75	
IP3 Grimes1991[25]	O^{2-}-O^{2-}	108	0.38	56.06	O^{2-}	-4.4	296.8	
	Ce^{4+}-O^{2-}	1984.2	0.3494	26.44	Ce^{4+}	-7.3	1957	
IP4 Vyas1998-1[35]	O^{2-}-O^{2-}	9547.92	0.2192	32	O^{2-}	-2.04	9.3	
	Ce^{4+}-O^{2-}	1809.68	0.3547	20.4	Ce^{4+}	-0.2	177.84	
IP5 Vyas1998-2[35]	O^{2-}-O^{2-}	9547.92	0.2192	32	O^{2-}	-2.04	10.3	
	Ce^{4+}-O^{2-}	2531.5	0.335	20.4	Ce^{4+}	-0.2	177.84	

IP6 Minervini1999[36]	O^{2-}-O^{2-}	9547.96	0.2192	32	O^{2-}	0.2	6.3	
	Ce^{4+}-O^{2-}	1809.68	0.3547	20.4	Ce^{4+}	-2.04	177.84	
IP7 Lewis1985b[37]	O^{2-}-O^{2-}	22764.3	0.149	43.83	O^{2-}	5.85	116	
	Ce^{4+}-O^{2-}	1013.6	0.3949	0	Ce^{4+}	-2.67	51	
IP8 Gotte2007[38]	O^{2-}-O^{2-}	9533.421	0.234	224.88	O^{2-}	-6.5667	1759.8	
	Ce^{4+}-O^{2-}	755.1311	0.429	0	Ce^{4+}	4.6475	43.451	
IP9 Walsh2011[39]	Buckingham + Lennard Jones + Morse				O^{2-}	-3.208967	72.071301	203400583.793525
					Ce^{4+}	-4.364996	117.728645	93291981.620729

Table S2. Several variants of Buckingham potentials for CeO_2 were parameterised based on different ρ in the Ce-O interactions. These potentials share the same Ce-Ce and O-O short-range interactions, C_6 coefficients, and relative ionic polarisabilities. The cut-offs (r_{max}) for all short-range interactions are set to 15 Å. All the pairwise potentials only include shell-shell interactions. The performance of these IPs in describing the properties of CeO_2 is shown in Figure S1. The parameters highlighted in **bold** compose the optimal Buckingham potential for CeO_2 (denoted as "*IP10a*" in the main text).

(a) Common parameters

$A_{O\text{-}O}$ (eV)	$\rho_{O\text{-}O}$ (Å)	$C_{6\ O\text{-}O}$ (eV Å^6)	$A_{Ce\text{-}Ce}$ (eV)	$\rho_{Ce\text{-}Ce}$ (Å)	$C_{6\ Ce\text{-}Ce}$ (eV Å^6)
**22764.30**	**0.1490**	**20.983768**	**1.0**	**0.1**	**30.481293**

(b) Variations

	$A_{Ce\text{-}O}$ (eV)	$\rho_{Ce\text{-}O}$ (Å)	$C_{6\ Ce\text{-}O}$ (eV Å^6)	Y_{Ce} (e)	Y_O (e)	$k_{2\ Ce}$ (eV Å^{-2})	$k_{2\ O}$ (eV Å^{-2})
IP S1	2348.8104	0.34	25.082349	20.05	-3.434	2594.0172	75.099753
IP S2	2155.1952	0.345	25.082349	18.7	-3.256	2247.4976	65.246179
IP10a	**2020.3688**	**0.348857**	**25.082349**	**15.040018**	**-3.297314**	**1382.9640**	**68.733447**
IP S3	1828.3487	0.355	25.082349	12.725902	-3.164543	935.88924	62.228793

IP S4	1690.1626	0.359995	25.082349	12.15	-3.044333	889.27773	54.218098
IP S5	1511.5856	0.367349	25.082349	10.05	-2.960088	580.31659	50.090402
IP S6	1453.5642	0.370001	25.082349	9.572565	-2.904296	518.46754	47.465871

Table S3. Atomic displacements due to the formation of intrinsic point defects in CeO_2 predicted by the M-L and QM/MM approaches. "-" and "+" indicate the displacement towards and away from the defect site, respectively.

	M-L		QM/MM B97-2		QM/MM PBE0		QM/MM BB1K	
	d_{O} (Å)	d_{Ce} (Å)	d_{O} (Å)	d_{Ce} (Å)	d_{O} (Å)	d_{Ce} (Å)	d_{O} (Å)	d_{Ce} (Å)
$\mathrm{V_O^{\bullet\bullet}}$	-0.275	+0.166	-0.245	+0.170	-0.237	0.174	-0.223	+0.180
$\mathrm{V_{Ce}''''}$	+0.431	-0.045	+0.296	-0.052	+0.291	-0.059	+0.301	-0.056
$\mathrm{O_i''}$	+0.162	-0.119	+0.173	-0.109	+0.164	-0.113	+0.159	-0.116
$\mathrm{Ce_i^{\bullet\bullet\bullet\bullet}}$	+0.096	+0.357	+0.084	+0.349	+0.080	+0.348	+0.066	+0.337

3 Interatomic potential parameters for CeO_2 (*IP10b*) in GULP-readable format

```
species
Ce4   core   -9.850000
Ce4   shel   13.850000
Ce3   core   -9.850000
Ce3   shel   12.850000
O2    core    0.936345
O2    shel   -2.936345
O1    core    0.936345
O1    shel   -1.936345

buck
O2    shel O2     shel   22764.300      0.149000   20.983768   0.000   15.00
O2    shel O1     shel   22764.300      0.149000   20.983768   0.000   15.00
O1    shel O1     shel   22764.300      0.149000   20.983768   0.000   15.00
O2    shel Ce4    shel   1138.963021    0.417578   25.082349   0.000   5.278
```

```
O2    shel Ce3   shel   1025.066719   0.417578   25.082349   0.000   5.532
O1    shel Ce4   shel   706.157073    0.417578   25.082349   0.000   5.278
Ce4   shel Ce4   shel   1.0000000     0.100000   30.481293   0.000   15.00
Ce3   shel Ce3   shel   1.0000000     0.100000   30.481293   0.000   15.00
Ce3   shel Ce4   shel   1.0000000     0.100000   30.481293   0.000   15.00

lennard 12   6
O2    shel O2    shel   1.0   0.0   0.000   15.00
O2    shel O1    shel   1.0   0.0   0.000   15.00
O1    shel O1    shel   1.0   0.0   0.000   15.00
O2    shel Ce4   shel   1.0   0.0   0.000   5.278
O2    shel Ce3   shel   1.0   0.0   0.000   5.532
O1    shel Ce4   shel   1.0   0.0   0.000   5.278
Ce4   shel Ce4   shel   1.0   0.0   0.000   15.00
Ce3   shel Ce3   shel   1.0   0.0   0.000   15.00
Ce3   shel Ce4   shel   1.0   0.0   0.000   15.00

morse
O2   shel   Ce4   shel -1.15172262   0.40   4.53327   0.000   5.278
O2   shel   Ce3   shel -1.036550358 0.40   4.53327   0.000   5.532
O1   shel   Ce4   shel -0.714068      0.40   4.53327   0.000   5.278

polynomial
1
O2   shel Ce4   shel   -1.0777670   0.000   0.000   5.278
polynomial
1
O2   shel Ce3   shel   -0.9249903   0.000   0.000   5.532
polynomial
1
O1   shel Ce4   shel   -0.7462155   0.000   0.000   5.278

spring
```

Ce4	1071.1845
Ce3	1071.1845
O2	53.022513
O1	53.022513

4 References

1. Blöchl, P. E., Projector augmented-wave method. *Phys. Rev. B* **1994,** *50* (24), 17953.
2. Perdew, J. P.; Burke, K.; Ernzerhof, M., Generalized gradient approximation made simple. *Phys. Rev. Lett.* **1996,** *77* (18), 3865.
3. Perdew, J. P.; Ruzsinszky, A.; Csonka, G. I.; Vydrov, O. A.; Scuseria, G. E.; Constantin, L. A.; Zhou, X.; Burke, K., Restoring the density-gradient expansion for exchange in solids and surfaces. *Phys. Rev. Lett.* **2008,** *100* (13), 136406.
4. Buckeridge, J.; Scanlon, D.; Walsh, A.; Catlow, C.; Sokol, A., Dynamical response and instability in ceria under lattice expansion. *Phys. Rev. B* **2013,** *87* (21), 214304.
5. Zhang, R.; Chutia, A.; Sokol, A. A.; Chadwick, D.; Catlow, C. R. A., A computational investigation of the adsorption of small copper clusters on the CeO2(110) surface. *Phys. Chem. Chem. Phys.* **2021,** *23* (35), 19329-19342.
6. Symington, A. R.; Molinari, M.; Moxon, S.; Flitcroft, J. M.; Sayle, D. C.; Parker, S. C., Strongly bound surface water affects the shape evolution of cerium oxide nanoparticles. *J. Phys. Chem. C* **2020,** *124* (6), 3577-3588.
7. Heyd, J.; Scuseria, G. E.; Ernzerhof, M., Hybrid functionals based on a screened Coulomb potential. *J. Chem. Phys.* **2003,** *118* (18), 8207-8215.
8. Adamo, C.; Barone, V., Toward reliable density functional methods without adjustable parameters: The PBE0 model. *J. Chem. Phys.* **1999,** *110* (13), 6158-6170.
9. Tasker, P., The stability of ionic crystal surfaces. *J. Phys. C: Solid State Phys.* **1979,** *12* (22), 4977.
10. Wardenga, H. F.; Klein, A., Surface potentials of (111),(110) and (100) oriented CeO2− x thin films. *Appl. Surf. Sci.* **2016,** *377*, 1-8.
11. Skorodumova, N.; Baudin, M.; Hermansson, K., Surface properties of CeO 2 from first principles. *Phys. Rev. B* **2004,** *69* (7), 075401.
12. Banerjee, A.; Adams, N.; Simons, J.; Shepard, R., Search for stationary points on surfaces. *J. Phys. Chem.* **1985,** *89* (1), 52-57.
13. Gale, J. D.; Rohl, A. L., The general utility lattice program (GULP). *Mol. Simul.* **2003,** *29* (5), 291-341.
14. Nolan, M.; Fearon, J. E.; Watson, G. W., Oxygen vacancy formation and migration in ceria. *Solid State Ionics* **2006,** *177* (35-36), 3069-3074.
15. Sun, L.; Huang, X.; Wang, L.; Janotti, A., Disentangling the role of small polarons and oxygen vacancies in Ce O 2. *Phys. Rev. B* **2017,** *95* (24), 245101.
16. Buckeridge, J.; Catlow, C. R. A.; Farrow, M.; Logsdail, A. J.; Scanlon, D.; Keal, T.; Sherwood, P.; Woodley, S.; Sokol, A.; Walsh, A., Deep vs shallow nature of oxygen vacancies and consequent n-type carrier concentrations in transparent conducting oxides. *Phys. Rev. Mater.* **2018,** *2* (5), 054604.
17. Press, W. H.; Teukolsky, S. A.; Vetterling, W. T.; Flannery, B. P., *Numerical Recipes 3rd Edition: The Art of Scientific Computing*. Cambridge University Press: 2007.
18. Sherwood, P.; de Vries, A. H.; Guest, M. F.; Schreckenbach, G.; Catlow, C. R. A.; French, S. A.; Sokol, A. A.;

Bromley, S. T.; Thiel, W.; Turner, A. J., QUASI: A general purpose implementation of the QM/MM approach and its application to problems in catalysis. *J. Mol. Struct.: THEOCHEM* **2003,** *632* (1-3), 1-28.
19. Valiev, M.; Bylaska, E. J.; Govind, N.; Kowalski, K.; Straatsma, T. P.; Van Dam, H. J.; Wang, D.; Nieplocha, J.; Apra, E.; Windus, T. L., NWChem: A comprehensive and scalable open-source solution for large scale molecular simulations. *Comput. Phys. Commun.* **2010,** *181* (9), 1477-1489.
20. Gulde, R.; Pollak, P.; Weigend, F., Error-balanced segmented contracted basis sets of double-ζ to quadruple-ζ valence quality for the lanthanides. *J. Chem. Theory Comput.* **2012,** *8* (11), 4062-4068.
21. Weigend, F.; Ahlrichs, R., Balanced basis sets of split valence, triple zeta valence and quadruple zeta valence quality for H to Rn: Design and assessment of accuracy. *Phys. Chem. Chem. Phys.* **2005,** *7* (18), 3297-3305.
22. Molinari, M.; Parker, S. C.; Sayle, D. C.; Islam, M. S., Water adsorption and its effect on the stability of low index stoichiometric and reduced surfaces of ceria. *J. Phys. Chem. C* **2012,** *116* (12), 7073-7082.
23. Huang, B.; Gillen, R.; Robertson, J., Study of CeO2 and its native defects by density functional theory with repulsive potential. *J. Phys. Chem. C* **2014,** *118* (42), 24248-24256.
24. Luo, S.; Li, M.; Fung, V.; Sumpter, B. G.; Liu, J.; Wu, Z.; Page, K., New Insights into the Bulk and Surface Defect Structures of Ceria Nanocrystals from Neutron Scattering Study. *Chem. Mater.* **2021,** *33* (11), 3959-3970.
25. Grimes, R.; Catlow, C. R. A., The stability of fission products in uranium dioxide. *Philos. Trans. R. Soc., A* **1991,** *335* (1639), 609-634.
26. Liu, X.-Y.; Sickafus, K. E., Lanthanum energetics in cubic ZrO2 and UO2 from DFT and DFT+ U studies. *J. Nucl. Mater.* **2011,** *414* (2), 217-220.
27. Steele, B. C. H.; Floyd, J. M.; Imperial Coll. of, S.; Tech, L., OXYGEN SELF-DIFFUSION AND ELECTRICAL TRANSPORT PROPERTIES OF NONSTOICHIOMETRIC CERIA AND CERIA SOLID SOLUTIONS. *Proc. Brit. Ceramic Soc. 19: 55-76(Mar 1971).* **1971**.
28. Fuda, K.; Kishio, K.; Yamauchi, S.; Fueki, K.; Onoda, Y., 17 O NMR study of Y2O3-doped CeO2. *J. Phys. Chem. Solids* **1984,** *45* (11-12), 1253-1257.
29. Park, D.; Griffith, J.; Nowick, A., Oxygen-ion conductivity and defect interactions in yttria-doped ceria. *Solid State Ionics* **1981,** *2* (2), 95-105.
30. Bredas, J.-L., Mind the gap! *Mater. Horiz.* **2014,** *1* (1), 17-19.
31. Freeman, C.; Catlow, C., A computer modeling study of defect and dopant states in SnO2. *J. Solid State Chem.* **1990,** *85* (1), 65-75.
32. Haynes, W. M., *CRC handbook of chemistry and physics*. CRC press: 2014.
33. Butler, V.; Catlow, C.; Fender, B.; Harding, J., Dopant ion radius and ionic conductivity in cerium dioxide. *Solid State Ionics* **1983,** *8* (2), 109-113.
34. Sayle, T. X. T.; Parker, S. C.; Catlow, C. R. A., The role of oxygen vacancies on ceria surfaces in the oxidation of carbon monoxide. *Surf. Sci.* **1994,** *316* (3), 329-336.
35. Vyas, S.; Grimes, R. W.; Gay, D. H.; Rohl, A. L., Structure, stability and morphology of stoichiometric ceria crystallites. *J. Chem. Soc., Faraday Trans.* **1998,** *94* (3), 427-434.
36. Minervini, L.; Zacate, M. O.; Grimes, R. W., Defect cluster formation in M2O3-doped CeO2. *Solid State Ionics* **1999,** *116* (3-4), 339-349.
37. Lewis, G.; Catlow, C., Potential models for ionic oxides. *J. Phys. C: Solid State Phys.* **1985,** *18* (6), 1149.
38. Gotte, A.; Spångberg, D.; Hermansson, K.; Baudin, M., Molecular dynamics study of oxygen self-diffusion in reduced CeO2. *Solid State Ionics* **2007,** *178* (25-26), 1421-1427.
39. Walsh, A.; Woodley, S. M.; Catlow, C. R. A.; Sokol, A. A., Potential energy landscapes for anion Frenkel-pair

formation in ceria and india. *Solid State Ionics* **2011,** *184* (1), 52-56.
